# Higher-Order Musical Temporal Structure in Bird Song

**DOI:** 10.3389/fpsyg.2021.629456

**Published:** 2021-03-25

**Authors:** Hans T. Bilger, Emily Vertosick, Andrew Vickers, Konrad Kaczmarek, Richard O. Prum

**Affiliations:** ^1^Department of Ecology and Evolutionary Biology, and Peabody Museum of Natural History, Yale University, New Haven, CT, United States; ^2^Department of Integrative Biology, University of Texas, Austin, TX, United States; ^3^Department of Epidemiology and Biostatistics, Memorial Sloan Kettering Cancer Center, New York, NY, United States; ^4^Department of Music, Yale University, New Haven, CT, United States

**Keywords:** bio-musicology, musicality, linguistics, bird song, sexual selection, honest signaling, perceptual bias, aesthetic evolution

## Abstract

Bird songs often display musical acoustic features such as tonal pitch selection, rhythmicity, and melodic contouring. We investigated higher-order musical temporal structure in bird song using an experimental method called “music scrambling” with human subjects. Recorded songs from a phylogenetically diverse group of 20 avian taxa were split into constituent elements (“notes” or “syllables”) and recombined in original and random order. Human subjects were asked to evaluate which version sounded more “musical” on a per-species basis. Species identity and stimulus treatment were concealed from subjects, and stimulus presentation order was randomized within and between taxa. Two recordings of human music were included as a control for attentiveness. Participants varied in their assessments of individual species musicality, but overall they were significantly more likely to rate bird songs with original temporal sequence as more musical than those with randomized temporal sequence. We discuss alternative hypotheses for the origins of avian musicality, including honest signaling, perceptual bias, and arbitrary aesthetic coevolution.

## Introduction

Many bird songs show striking behavioral, neural, genetic, and developmental parallels with human language (Doupe and Kuhl, 1999; Bolhuis et al., 2010; Bolhuis, 2013; Lipkind et al., 2013; Jarvis, 2019; Hyland Bruno et al., 2020). Like language, bird song can be combinatorial and hierarchical: Elements, or notes, combine to form syllables; syllables are combined into phrases; phrases are combined to form songs; and multiple songs comprise an individual song repertoire (ten Cate and Okanoya, 2012; Bowling and Fitch, 2015). But the linguistic analogy breaks down above this basic scheme. Since bird songs seem to lack symbolic meaning beyond basic functional reference, they cannot have words, semantics, or syntax in the strict linguistic sense (Marler et al., 1992; Berwick et al., 2011; Bowling and Fitch, 2015). To make empirical sense of the structure and content of bird song, we need different conceptual tools.

Music can be defined as “human sound communication outside the scope of spoken language” (Nettl, 2005:25). Although music varies greatly across cultures in its acoustic features, behavioral context, and conceptual framing, it also appears to have remarkable statistically “universal” qualities (Stumpf, 1911; Voss and Clarke, 1975; Nettl, 2005; Savage et al., 2015; Mehr et al., 2019). Analyzing a diverse global ethnographic sample, Mehr et al. (2019) found that human vocal songs: (1) showed more variation within than between societies, (2) were reliably associated with behavioral contexts like love, dance, infant care, religious activity, play, and healing, (3) nearly always displayed tonality, and (4) showed power-law distributions of melodic and rhythmic ratios, where a few frequently used ratios dominate, followed by a “heavy tail” of rarer ones (Adamic, 2011). Bird song has described in musical terms for centuries—in *Historia Animalium* (Stap, 2005), Aristotle described a nightingale giving “lessons in singing to a young bird”—but until recently, most scientific literature on music and bird song was biologically superficial and overly reliant on Eurocentric conceptions of musical structure (Rothenberg et al., 2014). Convergences between bird song and music were treated as objects of idle charm, and comparative studies often simply examined times when famous Western composers got inspired by birds (e.g., Keister and Baptista, 2005).

However, Darwin (1871), Craig (1943), Armstrong (1963), Hartshorne (1973) and Rothenberg et al. (2014) and others have given serious consideration to the idea that musical aesthetics can provide intellectual insights into avian evolution. Hartshorne (1958, 1973) considered bird song an “evolutionary anticipation of human music,” and used a six-dimensional rating system to quantify the “singing skill” of thousands of avian species according to the parameters: Loudness or carrying power, Scope (variety and complexity), Continuity (shortness of pauses in a standard performance), Tone quality (shown by narrow bands in a spectrogram), Organization or Order (Gestalt closure, musical coherence), and Imitative ability. Though Hartshorne's analyses were statistically rudimentary, his explicit goal was to relate “song-development” to biologically relevant factors such as behavioral context, plumage coloration, diet, and habitat (Hartshorne, 1973).

Recently, several more investigations of bird song musicality have been conducted with new empirical rigor (Fitch, 2015). Doolittle et al. (2014) and Araya-Salas (2012) compared the frequency ratios used in Hermit Thrush (*Catharus guttatus*) and Nightingale Wren (*Microcerculus philomela*) song to common harmonic intervals used in Western tonal music, demonstrating convergence in the first case and its absence in the second. Using human subjects, Doolittle and Brumm (2012) found that synthesized versions of Musician Wren (*Cyphorhinus arada*) songs that preserved the original intervallic relationships between notes evaluated as more “musical” than songs with slightly deformed tonal relationships. Patel et al. (2009; see also Keehn et al., 2019) reported behavioral evidence of musical beat perception and synchronization in a Sulfur-crested Cockatoo (*Cacatua galerita eleonora*), the first description of such behavior in a non-human animal. In another rhythmicity study, Roeske et al. (2020) showed that Thrush Nightingale (*Luscinia luscinia*) rhythms, similar to many human ones, are categorical and centered around small ratios. Earp and Maney (2012) found when female White-throated Sparrows (*Zonotrichia albicollis*) in a reproductive state listened to song from male conspecifics, immediate early gene activity increased in every region of the mesolimbic reward pathway that shows differential response to music in its putative human homolog.

Many musical signals also exhibit temporal structure at more complex levels of organization. Rothenberg et al. (2014) quantified aspects of “higher order” musicality by using phase and Wiener entropy plots to trace the rhythmic and tonal trajectories of Thrush Nightingale songs, uncovering “escalations” and “modifications” of rhythm and frequency formally similar to some human music. Studying the same species, Roeske et al. (2018) used “multi-fractal analysis” to uncover musical variations in the timing, duration, and intensity of notes across different levels of temporal hierarchy. Along similar lines, Janney et al. (2016) found that Pied Butcherbirds (*Cracticus nigrogularis*) with more “phrase types” in their repertoires tended to repeat common motifs shared across phrase types more often than those with repertoires composed of fewer phrase types. This implied a repertoire size-dependent optimization of “balance between repetition and novelty” in long song bouts (Janney et al., 2016).

Musical structure at these higher levels may exist in bird song, but the fact cannot be assumed a priori. Musicality is a percept—not a physically definable property of a sound—and humans are the only species from which we can collect direct reports on subjective aesthetic evaluations. So, the best way to test for the presence of musicality in bird song is to use humans as musical feature detectors. As one of the classic ethnomusicology texts states, “All humans can identify music—though not necessarily understand it—when they hear it” (Nettl, 2005:25).

Here, we used human subjects to evaluate whether there is higher-order, musical temporal structure in complex bird songs. By temporal musical structure, we mean time-based variation in acoustic content that elicits a positive hedonic response in the listener. To test for the existence of musical structure, we used the method “music scrambling” from the field of experimental music cognition (Levitin and Menon, 2003, 2005; Abrams et al., 2011), which involves reordering segments of recorded sound so that its temporal structure can be disturbed without significantly altering its global length or total spectral content. Functional MRI studies have shown differential responses to normal and scrambled music in music-sensitive populations of neurons in humans (e.g., Norman-Haignere et al., 2015).

To our knowledge, this is the first general test of the existence of high-order musical temporal structure across a range of avian taxa.

## Materials and Methods

### Sampling Method

We are not investigating whether all bird songs have high-order musical temporal structure, but whether any bird songs do. Thus, we used a specifically biased sample of highly complex bird songs exhibiting a variety of what we perceived to be musical features. We did not include in our sample any songs that obviously lack complex temporal structure among syllables, such as songs that include a series of identical notes uttered at a continuous pace (e.g., Chipping Sparrow, *Spizella passerina*). Rather, we selected songs characterized by complex acoustic structure composed of discrete and variable notes or syllables. We also selected bird songs with notes and syllables that were temporally discrete rather than graded and continuous in order to facilitate temporal scrambling without creating obvious acoustic artifacts.

Our sample included single songs or single song bouts from 20 bird species from 13 different families (Table 1). Nineteen samples were songs of male oscine songbirds (Passeri, Passeriformes), and one was a mechanically produced winnowing tail sound from a displaying male Wilson's Snipe (*Gallinago delicata*; Scolopacidae, Charadriiformes). Recordings were collected from various archival sources (see Table 1).

**Table 1:** Experimental stimulus identities and sources.

**Family**	**Common name**	**Species**	**Source**
Scolopacidae	Wilson's Snipe	*Gallinago delicata*	C
Atrichornidae	Noisy Scrubbird	*Atrichornis clamosus*	I
Acanthizidae	Scrubtit	*Acanthornis magna*	J
	Striated Fieldwren	*Calamanthus fuliginosis*	J
Artamidae	Gray Butcherbird	*Cracticus torquatus*	E
Pachycephalidae	Gray Shrikethrush	*Colluricincla harmonica*	E
Regulidae	Common Firecrest	*Regulus ignicapilla*	D
Cettidiae	Japanese Bush Warbler	*Horornis diphone*	F
Muscicapidae	Madagascar Magpie-Robin	*Copsychus albospecularis*	H
Mimidae	Brown Thrasher	*Toxostoma rufum*	B
Troglodytidae	Canyon Wren	*Catherpes mexicanus*	C
	Winter Wren	*Troglodytes hiemalis*	C
Fringillidae	Citril Finch	*Carduelis citrinella*	D
	Common Chaffinch	*Fringilla coelebs*	D
Passerellidae	Red Fox Sparrow	*Passerella iliaca*	B
	White-crowned Sparrow	*Zonotrichia leucophrys*	B
	Field Sparrow	*Spizella pusilla*	B
	Vesper Sparrow	*Pooecetes gramineus*	G
	Bachman's Sparrow	*Peucaea aestivalis*	A
Cardinalidae	Lazuli Bunting	*Passerina amoena*	G
**Attentiveness Control**	Fiddle	*Homo sapiens*	K
	Banjo	*Homo sapiens*	L

*Recording sources: A, Stokes Field Guide to Bird Songs: Eastern Region; B, Donald Kroodsma, The Singing Life of Birds; C, Cornell Lab of Ornithology, The Diversity of Animal Sounds; D, Andreas Schulze and Karl-Heinz Dingler, Die Vogelstimmen Europas, Nordafrikas, und Vorderasiens; E, David Stewart, Australian Bird Calls: Subtropical East; F, Hideo Ueda, Wild Bird Songs of Japan; G, Stokes Field Guide to Bird Songs: Western Region; H, British Library Sound Archive, Bird Sounds of Madagascar; I, XC40687, Mark Harper, xeno-canto.org; J, David Stewart, Australian Bird Calls: Tasmania; K, “Rolling Waves,” Pitnacree, unreleased recording; L, “Josie-O” on Earth Tones, by Adam Hurt*.

### Stimulus Preparation

High-quality digital audio files of bird songs with minimal background noise were edited using the program Audition CC (Adobe Audition CC, Adobe Systems, San Diego, CA, USA). First, each note or syllable was split into a separate audio file. Each file began at the exact onset of the sound and ended just before the onset of the next note or syllable (i.e., gaps between syllables were grouped with the previous syllable). Terminal syllables were cut off after the end of visually detectable sound in the spectrogram. Editing was done visually following ten Cate and Okanoya (2012). Thus, edited audio files varied in length with the length of the note or syllable from ~6 ms to longer than 1 s. Some recordings from species with comparatively short or fast-paced songs included multiple songs; in these cases, notes/syllables were randomized within individual songs and the periods of silence between songs were preserved. 2 s of relative silence from the original source recording was added before the beginning and after the ending of each song in order to normalize the presentation of stimuli. Envelopes of ~0.2 s were applied to each note/syllable file to reduce boundary artifacts upon recombination. Audition's spectral editing tool was used to decrease background noise, normalize the recordings and remove unwanted sonic artifacts (other bird vocalizations, environmental noise, etc.). The Noise Reduction tool was used to decrease the general background noise, and prominent artifacts were manually removed from the spectrogram.

**Figure 1:**
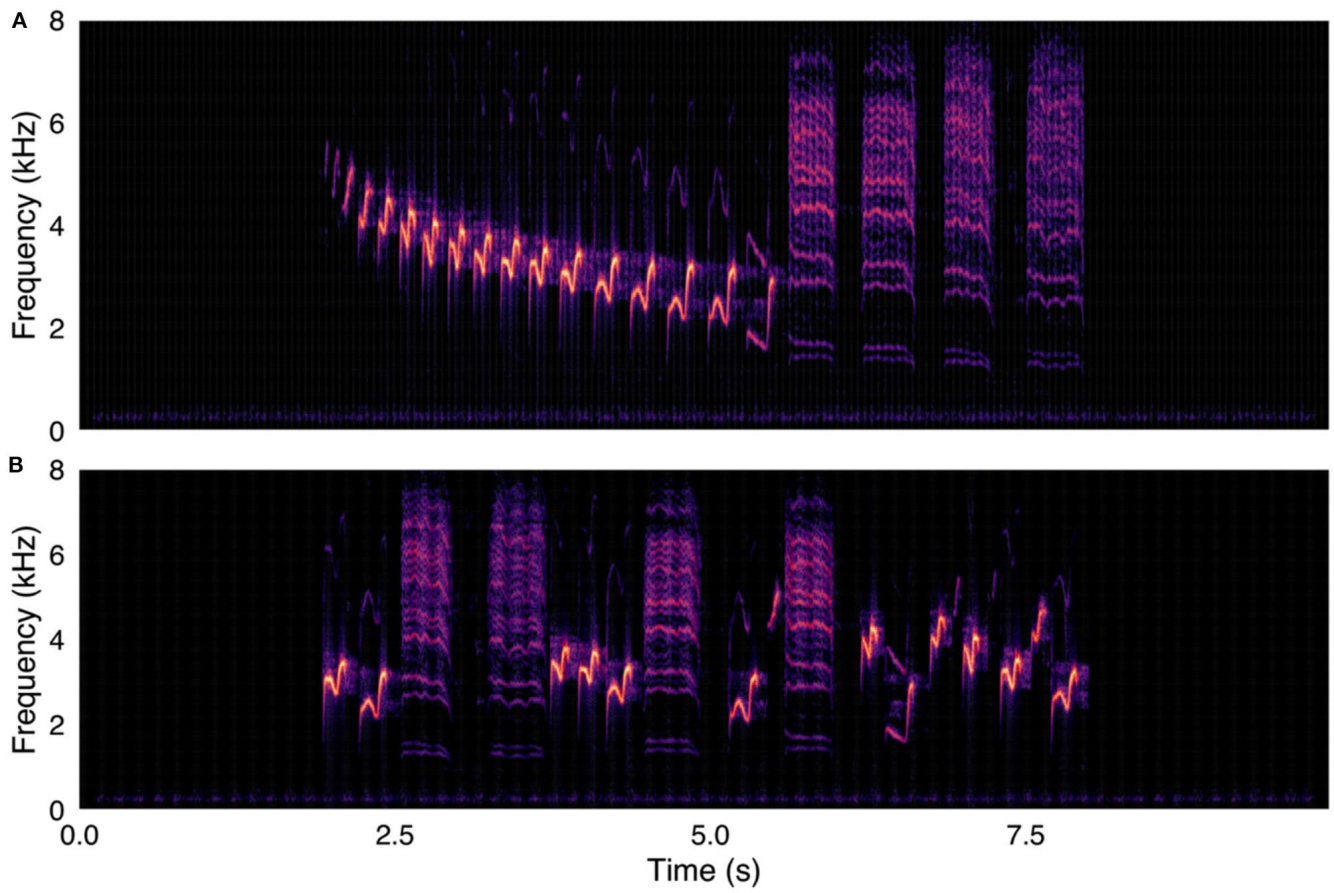
(**A**, upper panel) Canyon wren (*Catherpes mexicanus*) recording used in survey, with song elements in original temporal order. (**B**, lower panel) Same Canyon wren recording, with song elements in randomized temporal order. Spectrograms were created with were created using a 1024-point FFT and a Hamming window with 87.5% overlap.

**Figure 2:**
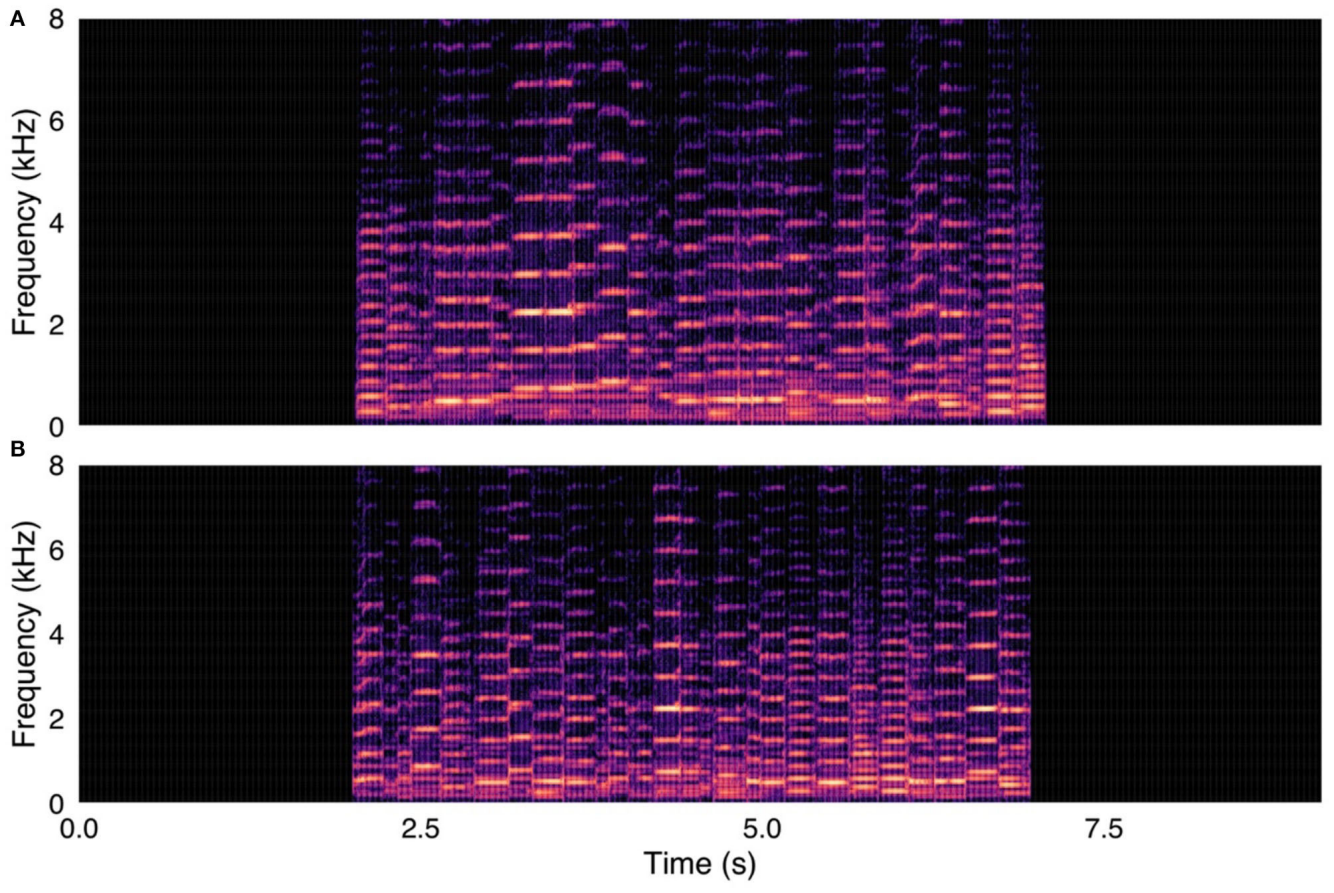
(**A**, upper panel) Control stimulus from survey. Human fiddle music recording, with notes in original temporal order. (**B**, lower panel) Same fiddle music recording, with notes in randomized temporal order. Spectrograms were created with were created using a 1024-point FFT and a Hamming window with 87.5% overlap.

After each bird song was edited into its component notes/syllables, the edited audio files were recombined into two versions: one in original temporal order, and another with random temporal order (Figures 1, 2). The new recordings were then reviewed once more and converted to mp3 files for uploading onto the online survey platform. All stimulus audio files have been uploaded to Mendeley Data (doi: 10.17632/pkrvf77by8.2). Spectrograms of all stimuli are available in the Supplementary Material.

### Survey Design and Implementation

A psychophysical survey for human subjects was created using the online platform Qualtrics. In the survey, subjects were given the following prompt and instructions:

“Music is often characterized by tunefulness, harmony, temporal patterning, and thematic development. In each question, you will hear a series of two audio recordings. Please indicate which sounds more musical with regard to the qualities listed above.”

The prompt was designed to suggest to the subjects a broad range of criteria that might influence the evaluation of musicality without specifying temporal pattern exclusively. The subjects were then presented with original sequence and temporally randomized recordings from the 20 bird species under review. Two recordings of human music (excerpts of solo fiddle and banjo performances) were manipulated in a similar manner and presented along with the bird song recordings as a control for subject attentiveness or perverse responses. The order of species/human music presentation was randomized, as was the order of natural vs. manipulated stimuli within species. Subjects were required to start the playback of each recording themselves, and a timer was implemented so that it would not be possible to advance to the next sample until the subject had had time to listen to the entire recording.

All subjects provided personal demographic information including: gender; whether they were hearing impaired; whether they had experience identifying wild birds by song; whether they had ever owned pet birds; and how much prior musical experience they had. All subjects were 18 years or older. Following Doolittle and Brumm (2012), the musical experience categories available to participants were:

a.) “little or no experience studying music, singing, or playing an instrument,”
b.) “amateur/some experience studying music, singing, or playing an instrument,” and
c.) “professional/extensive experience studying music, singing, or playing an instrument.”

Summaries of subject demographic and personal experience data are presented in Table 2.

**Table 2:** Characteristics of survey subjects, *N* = 92.

Male gender	51 (55%)
Survey taker is hearing impaired	1 (1.1%)
Experience with studying music, singing, playing an instrument	
Little/no experience	42 (46%)
Amateur/some experience	46 (50%)
Professional/extensive experience	4 (4.3%)
Survey taker has experience with wild bird song	10 (11%)
Survey taker is a bird owner	27 (29%)

Subjects were recruited using Amazon's crowdsourcing marketplace Mechanical Turk, and paid a small fee for their participation. We only accepted subjects who had had at least 90% of their previous MTurk tasks approved, and IP address and geolocation information were used to ensure that duplicate surveys were not counted. On the MTurk website, the activity was entitled “Sound Musicality Survey.” No information provided to the participants mentioned that the sounds were bird songs. The survey took an average of 19 min and 56 s to complete.

### Statistical Analysis

We recruited a sample of 126 human subjects. There were 33 subjects who identified either the temporally randomized fiddle or banjo music as more musical than the original temporal sequence recordings. We concluded that they were inattentive or malicious, and they were eliminated from any further consideration (Fleischer et al., 2015). Given the frequency of inattentive responses to our two control questions, we could expect there may be as many as 23 additional inattentive subjects making random choices included in our analysis. One additional observation was excluded because it was a duplicate survey from the same coordinates (latitude and longitude); we kept the first survey taken from that respondent and excluded the second. The processed survey response dataset used in the statistical analysis can be found at the Mendeley Data link provided in Data Availability Statement.

We first assessed whether there was a difference in musicality ratings between the 20 species studied. Each participant evaluated all 20 species, leading to correlation between responses for different species assessed by the same participant. To account for the correlation in musicality evaluations between different species assessed by the same participant, we analyzed the responses from the remaining 92 subjects using a multilevel mixed-effects logistic regression model with the endpoint of correct assignment of original order vs. the endpoint of temporally randomized bird song. Species was included as a fixed effect in the model, participant as a random effect, with musical experience and bird ownership as covariates. To assess whether original order bird songs were more likely to be assessed as more musical by participants overall, we performed a meta-analysis using the proportion of participants choosing original order bird song across all species. All analyses were conducted using Stata 15 (Stata Corp., College Station, TX).

## Results

**Figure 3:**
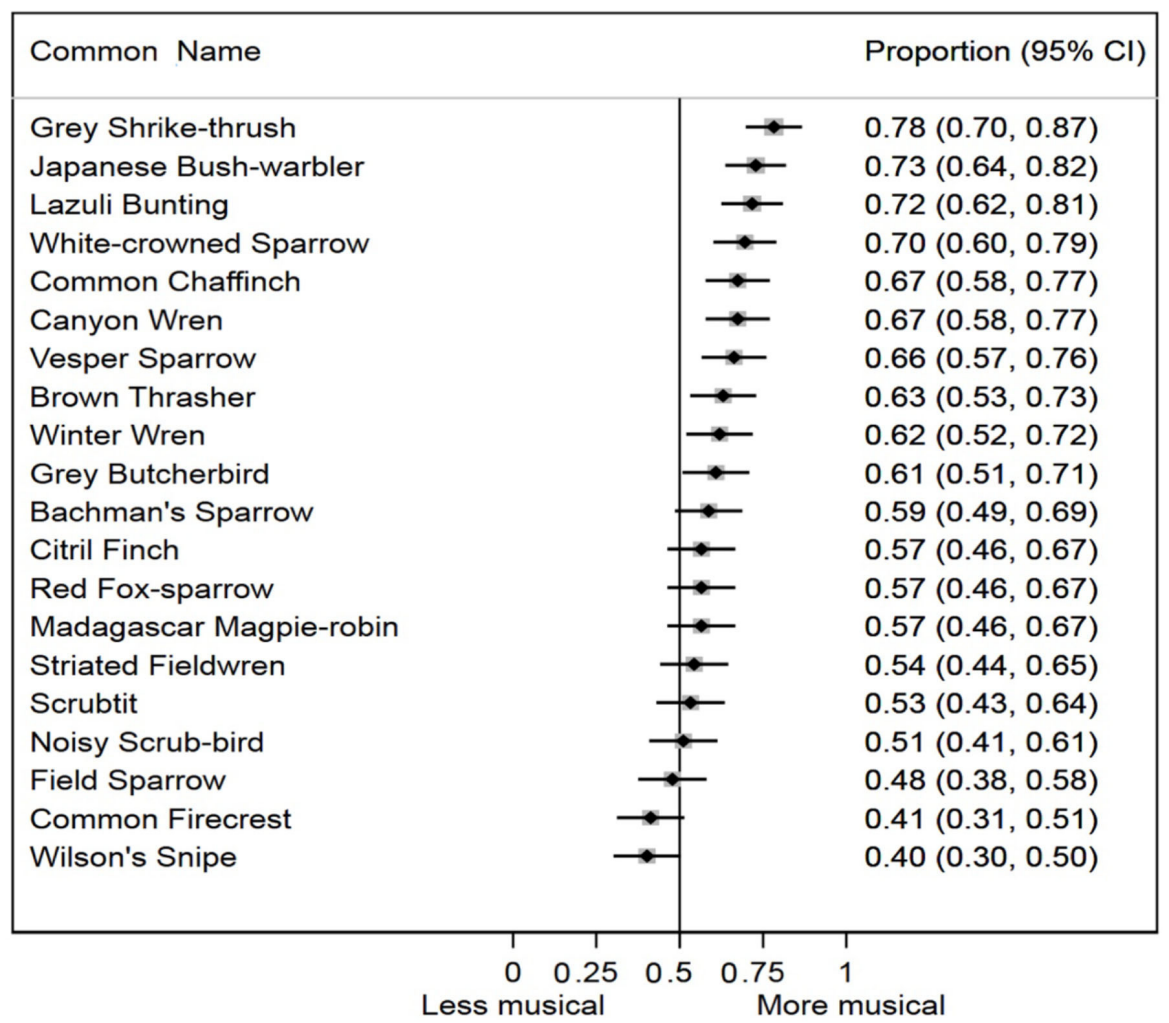
Proportion of human subjects who rated the original order song of each species as more musical, with 95% confidence intervals ordered from high to low.

The random effect on our logistic regression model was highly significant (*p* < 0.0001), suggesting that subjects varied in their musicality ratings of original order vs. temporally randomized songs of this sample of avian species. We also found a highly significant difference in musicality ratings across all bird species studied (*p* < 0.0001). For 10 of 20 species in the study, the 95% confidence intervals for the average musicality responses both excluded, and were more musical than, the null expectation (Figure 3). For seven of 20 species, the average musicality responses were more musical than, but did not exclude, the null. Only three of 20 species (Field Sparrow, Common Firecrest, and Wilson's Snipe) had average musicality evaluation responses that were less musical than the null expectation, but none of these were statistically distinguishable from the null. Combining the results from each species meta-analytically, bird songs with original temporal sequence were significantly more likely to be evaluated as more musical by human subjects than bird songs with randomized temporal sequence (*p* < 0.0001).

Musicality ratings were not affected by either musical experience of the subjects (some experience vs. no experience odds ratio 0.93; 95% C.I. 0.69, 1.26; extensive experience vs. no experience odds ratio 0.68; 95% C.I. 0.33, 1.40; *p* = 0.6) or by history of pet bird ownership (odds ratio 1.36; 95% C.I. 0.98, 1.88; *p* = 0.064).

## Discussion

We hypothesized that some acoustically complex bird songs have higher order, musical temporal structure. In other words, we hypothesize that syllable sequence is not temporally arbitrary for some birds, but has specifically evolved because of its aesthetic, musical impact upon the receiver. Given that the critical agents in this evolutionary process are birds themselves, it is reasonable to ask why our question was not addressed via behavioral tests on avian subjects. However, our question was whether bird song evinces high-order musical temporal structure at all. This is a subjective question that cannot be answered via behavioral observation. It requires “introspective access” (*sensu* Bowling and Fitch, 2015) to aesthetic evaluations, which means that only humans can confirm the broad existence of musical structure *per se*. So, we have used human subjects to evaluate the relative musicality of the specific, original sequence of notes/syllables found in natural songs compared to a randomized sequence of the same notes/syllables. We found strong support for the hypothesis that the temporal sequence of notes/syllables is musically non-random in some bird songs.

Western classical compositions are often characterized as having temporal development, such as introduction, variations on a theme, and creation/resolution of expectations (e.g., Huron, 2006; Morgan et al., 2019). Human psychological research demonstrates that perception of musicality is strongly associated with the presence of natural temporal structure (e.g., Norman-Haignere et al., 2015). This finding supports our conclusion that human subjects can perceive aesthetic temporal structure in some bird songs.

Bird songs are so much shorter in duration than most human musical performances, they may be better compared to musical motives (Hartshorne, 1973). Motives are shorter musical themes or “ideas” that are characterized by a series of notes of particular pitches and rhythmic sequence (Zbikowski, 1999). Motives also have aesthetic structure—i.e., their musical effect would be transformed or disrupted if the sequence of pitches or the amount of time between discrete sonic events were scrambled.

Some examples of musical temporal structure in bird song appear obvious to many listeners. Pied Butcherbirds sing in antiphonal choruses whose interlocking parts resemble the “hocketed” lines of medieval motets and freely improvised jazz (Taylor, 2010). Male Club-winged Manakins (*Machaeropterus deliciosus*) stridulate their secondary wing feathers during courtship to create sustained, string instrument-like tones (Bostwick and Prum, 2005). Among the species used in our sample, the songs of male Japanese Bush Warbler (*Horornis diphone*) are famous for their musicality (Hartshorne, 1973). They are characterized by an initial penetrating pure tone or series of tones that increase in volume, and an abrupt cascade of diverse and rapidly modulated pure tones on different frequencies. This temporal sequence of events has a clear analog in the “introduction, variation, and resolution” phrase structure found across many human musical traditions. Perhaps unsurprisingly, human subjects rated the natural song of Japanese Bush Warbler more musical at a higher frequency than nearly every other species in our sample.

When we scramble music, bird song, or speech, what exactly is disrupted for the receiver? In human speech, temporal patterning affects categorical perception. In certain cases, identical sounds are classified as different phonemes depending on their location in the speech stream (Bloch, 1941; Lachlan and Nowicki, 2015). Auditory perception in some songbirds exhibits a similar dependency. Lachlan and Nowicki (2015) studied three related note types in Swamp Sparrow (*Melospiza georgiana*) song, which they named “short,” “intermediate,” and “long.” The types are easy to separate via acoustic clustering methods, but the study population of Swamp Sparrows perceived only two categories in playback experiments: short and long. In further experiments, the authors determined that “intermediate” notes tended to be classified as “short” if they fell at the beginning of a syllable and “long” if they fell at the end. Roeske et al.'s (2020) discovery of small ratio, categorical rhythms in Thrush Nightingale songs suggests a similar perceptual grounding—scrambling note order redistributes the onset-to-onset interval times in a given song, possibly shifting them toward ratios less typical (and therefore possibly less preferred) for the species.

Above the level of segment sequence in speech is “prosody,” or variation in the frequency, timing, and intensity of elements over the course of a spoken phrase (Mol et al., 2017). Analogous variation in musical phrasing is sometimes called “musical prosody” and can be critical in establishing and manipulating musical expectation in listeners (Huron, 2006; Palmer and Hutchins, 2006; Heffner and Slevc, 2015). Certain songbirds appear sensitive to prosodic cues in human speech as well. In a set of behavioral experiments, Spierings and ten Cate (2014) found that Zebra Finches (*Taeniopygia guttata*) responded more to prosodic cues in manipulated human speech recordings than syntactic structure—and they responded even more strongly to prosody than human subjects did. Prosodic structure could be a productive study object for vocal evolution research since it is hierarchically organized, common to language and music, and independent from semantic meaning (Mol et al., 2017).

The fact that both birds and humans exhibit temporal pattern-dependent categorical perception and prosody salience is likely due to convergent evolution. But avian possession of these important perceptual building blocks of human musicality suggests that aesthetic perception of sound is likely not restricted to humans.

### Origins of Avian Musicality

Why should temporal musicality evolve in bird song? Unlike alarm calls, for example, bird song functions in sexual signaling, and is hypothesized to evolve primarily under sexual selection. We will consider three hypotheses concerning avian acoustic signal evolution: honest signaling theory, sensory/cognitive bias, and arbitrary aesthetic coevolution.

Honest signaling theory suggests that reliable information about signaler quality or condition is insured by the production and survival costs of the signal (Gil and Gahr, 2002). In honest signaling, the adaptive advantage of the song is an extrinsic property that is correlated with, but not part of, the sexual signal such as good genes that will enhance the survival of offspring, material resources necessary for reproduction, minimizing search costs, or protection from sexually transmitted diseases or other infections. In bird song, the potential for encoding information about mate quality in song has been hypothesized to be related to motor constraints, such as the trade-off between trill rate and frequency bandwidth in songbird song elements (e.g., Podos, 1997; Ballentine et al., 2004). However, in general, musical structures do not appear to be strongly shaped by such constraints. Human vocal songs tend to be “dominated by small melodic intervals and simple rhythmic ratios” (Mehr et al., 2019). A small melodic ratio implies a narrow frequency bandwidth between adjacent notes. If human songs were optimized for “performance,” we would expect them to compensate for these small ratios by favoring fast trills by design, at least by the trill rate/bandwidth interpretation of vocal performance. This is plainly untrue, as human song incorporates a variety of speeds and rhythms. Since human song appears to strongly deviate from vocal motor limits, it is “low performance” by design. Therefore, the presence of musical structure in bird song is not predicted by the most prominent avian acoustic application of honest signaling theory.

A second hypothesis is that sensory/cognitive biases emerge in the context of mate choice which arise from independent adaptations or basic design constraints of the sensory and cognitive systems (Ryan and Cummings, 2013; Renoult and Mendelson, 2019). A classic, acoustic example comes from the túngara frog (*Physalaemus pustulosus*), where the dominant frequencies of male advertisement call components match the previously-evolved tuning of the female inner ear organs (Ryan, 1985; Ryan and Rand, 1990; Ryan et al., 2019). Other biases may be more cognitively rooted, such as the preferences for more complex songs and/or larger repertoires in many oscines, possibly due to an adaptive avoidance of neurological habituation (e.g., Catchpole, 1986; Eda-Fujiwara et al., 2006; Ryan and Cummings, 2013). Importantly, though, evolution of traits due to sensory/cognitive biases alone will not lead to coevolution of traits/preferences. This is because such biases, by definition, are the result of natural selection on unrelated traits.

Alternatively, temporal musical structure in bird song could evolve because it is more aesthetically attractive to learners and receivers than other possible sonic sequences. Aesthetic coevolution involves sensory perception, cognitive evaluation, and choice based on genetically or culturally transmitted variation (Prum, 2012, 2017). More specifically, musical temporal structure in bird songs could evolve as an arbitrary sexually selected trait (Fisher, 1958; Lande, 1981; Kirkpatrick, 1982; Prum, 2010, 2012, 2017; Bailey and Moore, 2012)— i.e., there is no causal correlation between temporal musicality in bird song and signaler quality or condition. Such songs are neither honest nor dishonest because they are unrelated to any extrinsic quality information that can be lied about. Rather, they are merely available for aesthetic evaluation by receivers, and subject to subsequent sexual or social selection. However, the sharing of components of musicality by some complex bird songs and human music implies that these avian acoustic signals are extremely non-random in another way—their aesthetic impact upon the receiver (Rothenberg et al., 2014; Roeske et al., 2020).

To distinguish between arbitrary aesthetic coevolution (Bailey and Moore, 2012; Prum, 2012, 2017) and perceptual bias (Ryan and Cummings, 2013; Renoult and Mendelson, 2019), we need evidence of coevolution of preferences and traits. Evidence suggesting such a dynamic is abundant for oscine birds. Classic studies of male neural/cognitive templates, or learning biases, demonstrate that heritable, biologically evolving neural preferences can coevolve with the vocal structure of male song (e.g., Nottebohm, 1968, 1970; Marler and Waser, 1977; Marler and Sherman, 1983; Lachlan and Feldman, 2003). On the other extreme, Derryberry (2007) conducted a playback experiment on the White-crowned Sparrows (*Zonotrichia leucophrys*)—a species with a song that was ranked as having with among the most musical temporal structure in our sample (Figure 3). In a population in the Sierra Nevada, California, Derryberry played song recordings to wild female and male sparrows of contemporaneous male songs, and male songs recorded 24 years earlier at the same locality. She found that the older songs elicited nearly half the social response—either male territorial challenges, or curious female interest—as the contemporaneous songs did. In other words, cultural evolution in male White-crowned Sparrow song was associated with corresponding cultural coevolution in the social salience and attractiveness of those social signals. These data cannot be explained by a sensory bias alone.

However, the ability of both humans (in our experiments) and birds (avian evaluators in wild populations of these species) to perceive and prefer musical temporal structures in bird songs does imply the independent evolution of some broad cognitive preference for temporal aesthetic structure has evolved convergently in multiple different lineages of organisms, minimally including oscine birds and humans. This aesthetic concept could be defined as a kind of broad, non-adaptive, aesthetic cognitive bias—specifically a bias toward being aesthetically engaged by the attraction of attention, and the building and fulfillment of expectation. In this way, we can conceptualize the evolution of temporal musicality in bird song as the result of an interaction between arbitrary sexual selection and broad cognitive biases for aesthetically attractive temporal structure in acoustic sexual signals. Multiple lineages of oscine birds have independently evolved songs with higher-order musical temporal structure as they reached a certain threshold of acoustic complexity and strength of selection (Devoogd et al., 1993). Future work on aesthetic evolution in bird song should work to characterize the nature of acoustic aesthetic biases by comparing multiple evolutionary origins of complex, higher-order temporal structure to close relatives that lack such complexity.

Our explicitly aesthetic hypothesis for the evolution of bird song's musicality continues the tradition of Darwin (1871), who characterized male oscine song as “having the power to charm the female.” A century later, Hartshorne (1973) proposed the “monotony threshold hypothesis,” which proposes that vocal repertoire diversity evolves to prevent habituation—i.e., boredom—in the receiver. The monotony threshold suggests an inverse relationship between “continuity” and “variety” in bird song. Hartshorne posited that birds with more elaborate repertoires tend to sing more continuously than birds with simpler, more repetitive vocalizations. Vocal learning plays a role here as well: species that learn their songs tend to have larger repertoires than those who do not (Marler, 2000).

### Variation in Musical Temporal Structure Among Species

Empirical data already document that there are significant variations among species in bird song's musical acoustic structure. Some species appear to explore harmonic content, whereas others explore rhythmic variation (e.g., Doolittle et al., 2014; Rothenberg et al., 2014; Roeske et al., 2020). Although our study was not designed to investigate differential musical temporal structure among species, our data do yield some useful comparative observations. The four species with the highest perceived musical temporal structure in our study were Japanese Bush Warbler (*Horornis diphone*), Gray Shrikethrush (*Colluricincla harmonica*), Lazuli Bunting (*Passerina amoena*), and White-crowned Sparrow (*Zonotrichia leucophrys*). The songs of the latter three were complex along Hartshorne's (1973) dimensions of “singing skill,” described above for the Japanese Bush Warbler—they employed a variety of pure tone and broadband frequency elements, used a high degree of rhythmic variation, and had, to our ears, a sense of musical “development” and “resolution” over the course of an individual song.

The temporal complexity of these “highly musical” songs stood in noticeable contrast to the temporal structure of the songs of the three “least musical” species in our study—Field Sparrow (*Spizella pusilla*), Common Firecrest (*Regulus ignicapilla*), and Wilson's Snipe (*Gallinago delicata*). All three species have songs with a clear sense of rhythmic acceleration (or accelerando in Western musical terms), but they had much less spectral diversity. They were selected for the sample because they might manifest some minimal criteria for musical temporal structure. The Field Sparrow song begins with a rhythmic diminution, but its terminal trill simply repeats the same downward-sweeping note. A similar musical simplicity characterized the song of the Common Firecrest, which consists of a single repeated rhythm accelerating toward the end, with a rise in pitch as its only harmonic development. Wilson's Snipe received the lowest marks for musicality of any species, though this may be due to a unique interaction between the idiosyncrasies of its winnowing tail feather song and the nature of our acoustic manipulation. The song employs a steadily accelerating pulse of notes and features a bell curve-like pitch and loudness contour. When the notes were temporally randomized, however, this smooth progression of pitches and volumes was transformed into something that sounded far more syncopated (almost funky) to us, and apparently more musical to many human observers. In this case, it seems possible that our random manipulation created interesting musical temporal structure instead of breaking it down. Of course, the failure of these songs to be ranked as highly musical in our scrambling experiment does not mean that they are not actually examples of musical temporal structure. An alternative experiment could compare these natural songs to edited recordings of the same notes in the same temporal sequence but at a single, consistent tempo. A negative control (i.e., two alternative random orders of the same non-musical notes) could also be useful for future studies.

These examples also do not imply that bird song always sounds more musical as it gets more complex. The song of the Winter Wren (*Troglodytes troglodytes*) is an intricate stream of varied sounds and rhythms, but it received middling musicality scores in our study. The vagaries of individual aesthetic preference and differences in acoustic perception between humans and birds (such as the fact that birds can discern changes in the temporal structure of harmonic sounds at a much finer timescale than humans) make it impossible to render objective comparative judgements about avian musicality across species based on acoustic features alone (Hartshorne, 1973; Lohr et al., 2006). The likely presence of systemic biases in the musical preferences of our study subjects—who were by definition tech-savvy computer owners—underscores this point. In future studies, introducing a control featuring human vocal recordings (which are maximally analogous to bird song in terms of biomechanical production) could further validate the experimental method.

We think that human evaluations of bird musicality are scientifically informative. Indeed, if it were not for the aesthetic evaluation by humans of other species' display traits, the field of sexual selection would not exist at all. Darwin became “sick” at the sight of an eyespot on a Peacock's tail feather because he found it uselessly beautiful (Burkhardt et al., 1995). If the tail had not appeared beautiful to him, there would have been no need to seek a novel evolutionary mechanism to explain its visual aesthetics. Musical beauty is no different. Although there are clearly limits to the observational potential of human evaluators, using humans to establish the existence of high-order musical temporal structure in bird song is within these bounds, and effective.

## Data Availability Statement

All stimuli audio files and processed survey results are available for download at Mendeley Data (doi: 10.17632/pkrvf77by8.2 ).

## Ethics Statement

The studies involving human participants were reviewed by the Yale University Human Subjects Committee, and were granted an exemption from permit and informed consent requirements.

## Author Contributions

RP and HB conceived of the study and wrote the manuscript. HB created the stimuli, designed the psychophysical survey, and collected the data. EV and AV carried out the statistical analyses. KK designed and wrote Bird Call, the Max MSP patch used to recombine bird song notes/syllables. All authors contributed to the article and approved the submitted version.

## Conflict of Interest

The authors declare that the research was conducted in the absence of any commercial or financial relationships that could be construed as a potential conflict of interest.
