# Supplementary material for: Higher-Order Musical Temporal Structure in Bird Song
Source: Front Psychol. 2021 Mar 25;12:629456. doi: 10.3389/fpsyg.2021.629456 (PMC8044833; doi:10.3389/fpsyg.2021.629456)
Supplement: Supplementary file 1 [file Image_1.pdf]

Supplementary Material

1 Supplementary Figures

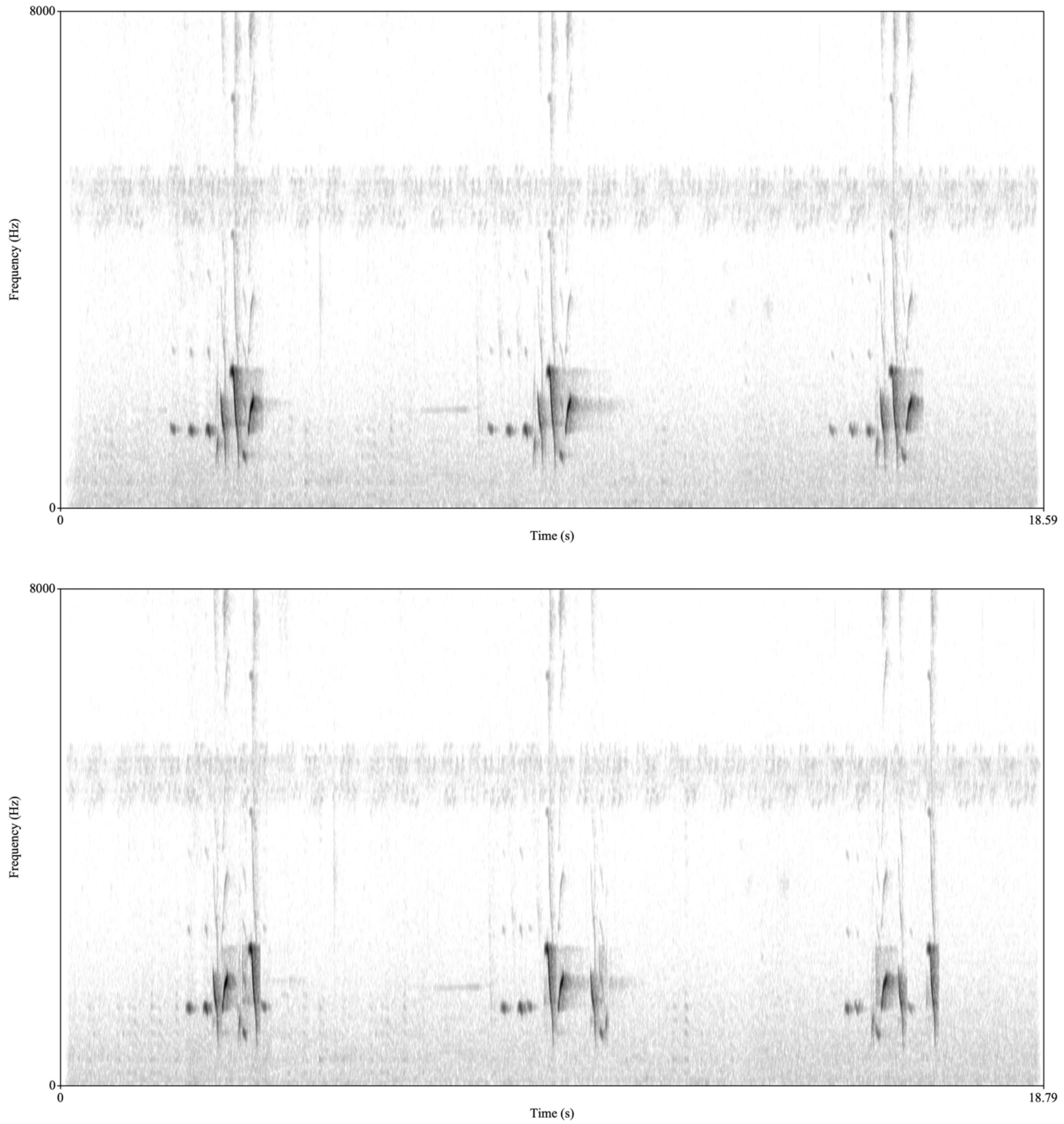

Supplementary Figure 1. Spectrogram representation of Grey Shrike-thrush (*Colluricincla harmonica*) stimuli. Top, original element order; bottom, randomized element order.

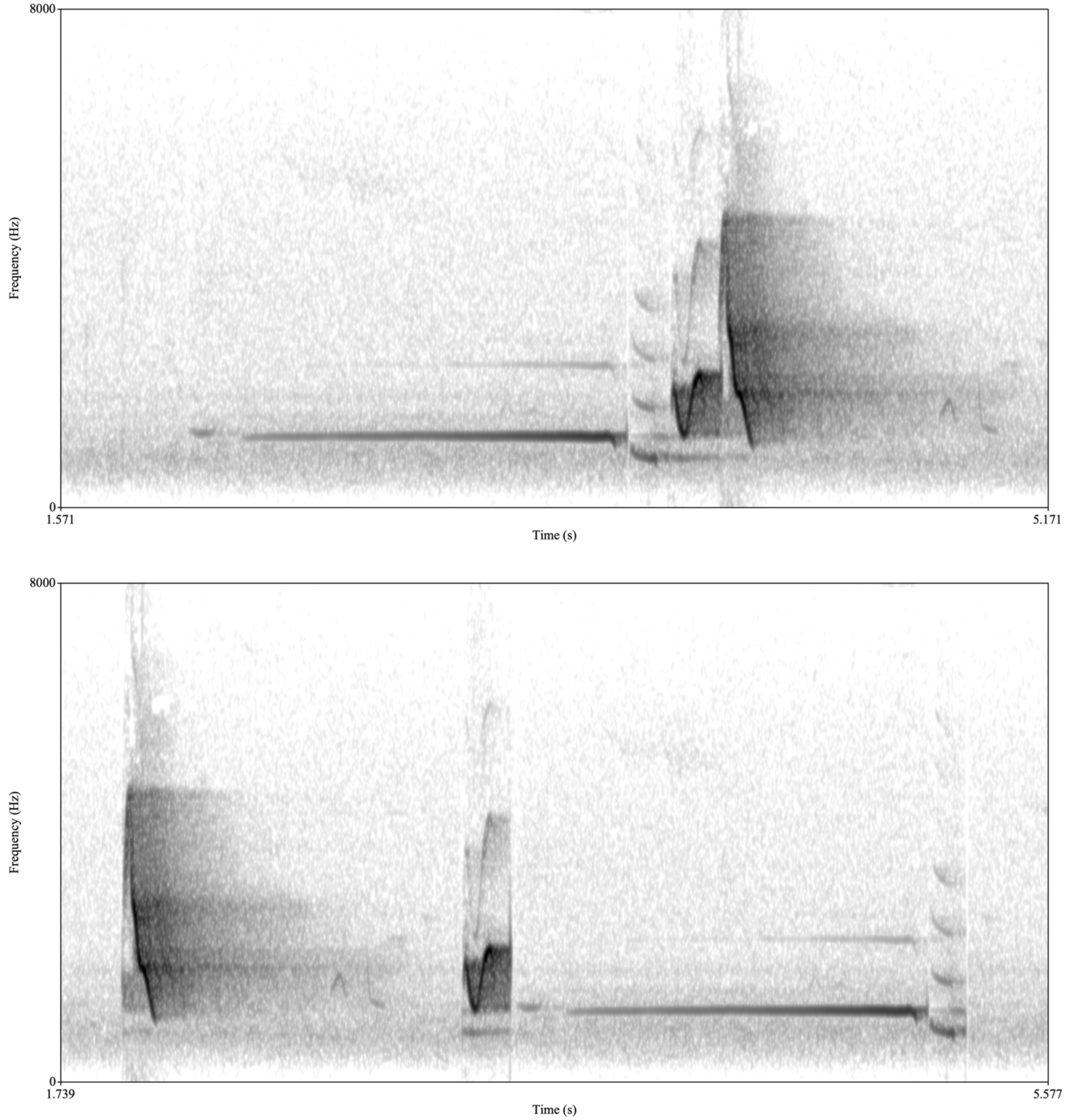

Supplementary Figure 2. Spectrogram representation of Japanese Bush-warbler (*Horornis diphone*) stimuli. This was the first of four songs used in the study stimulus. Top, original element order; bottom, randomized element order.

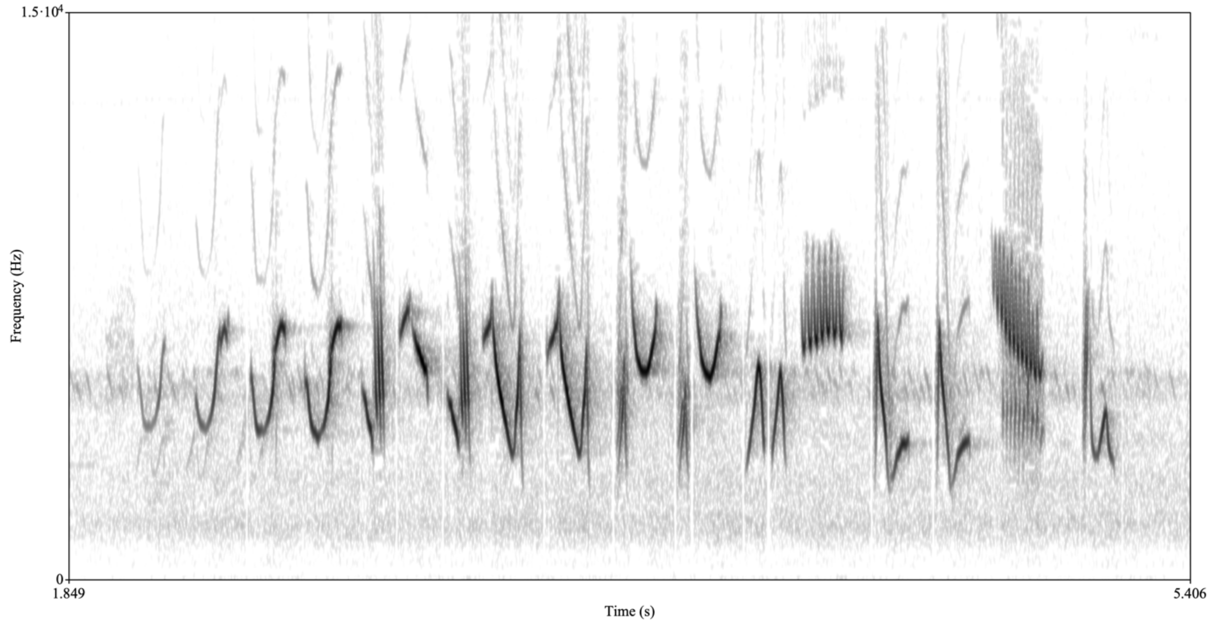

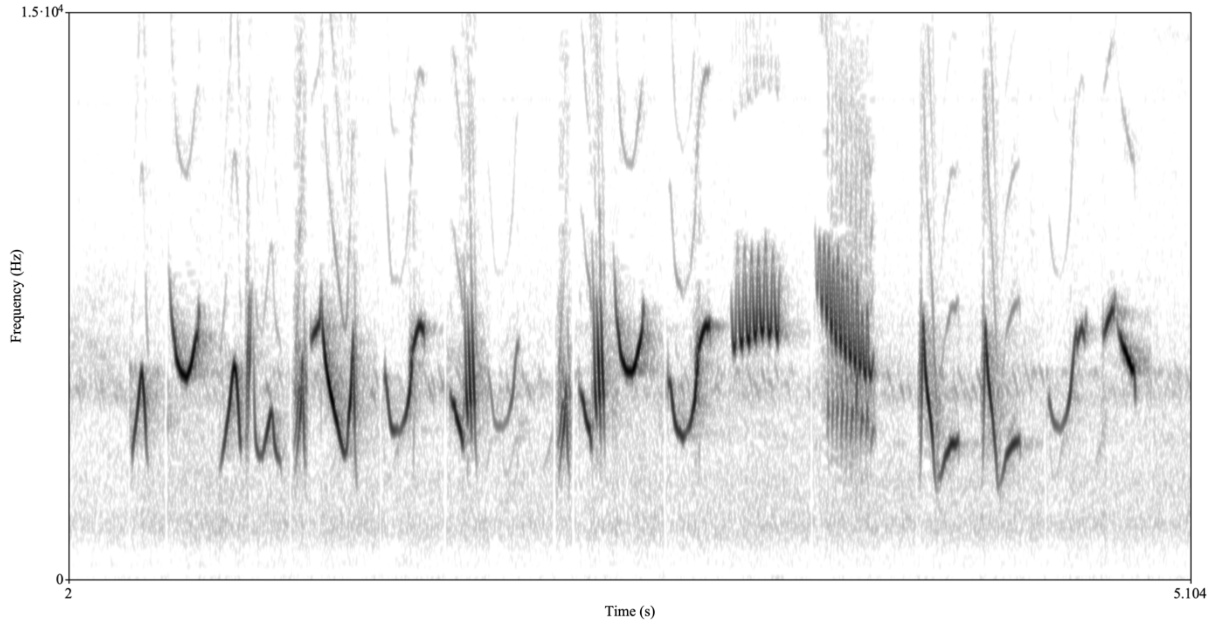

Supplementary Figure 3. Spectrogram representation of Lazuli Bunting (*Passerina amoena*) stimuli. This was the first of three songs used in the study stimulus. Top, original element order; bottom, randomized element order.

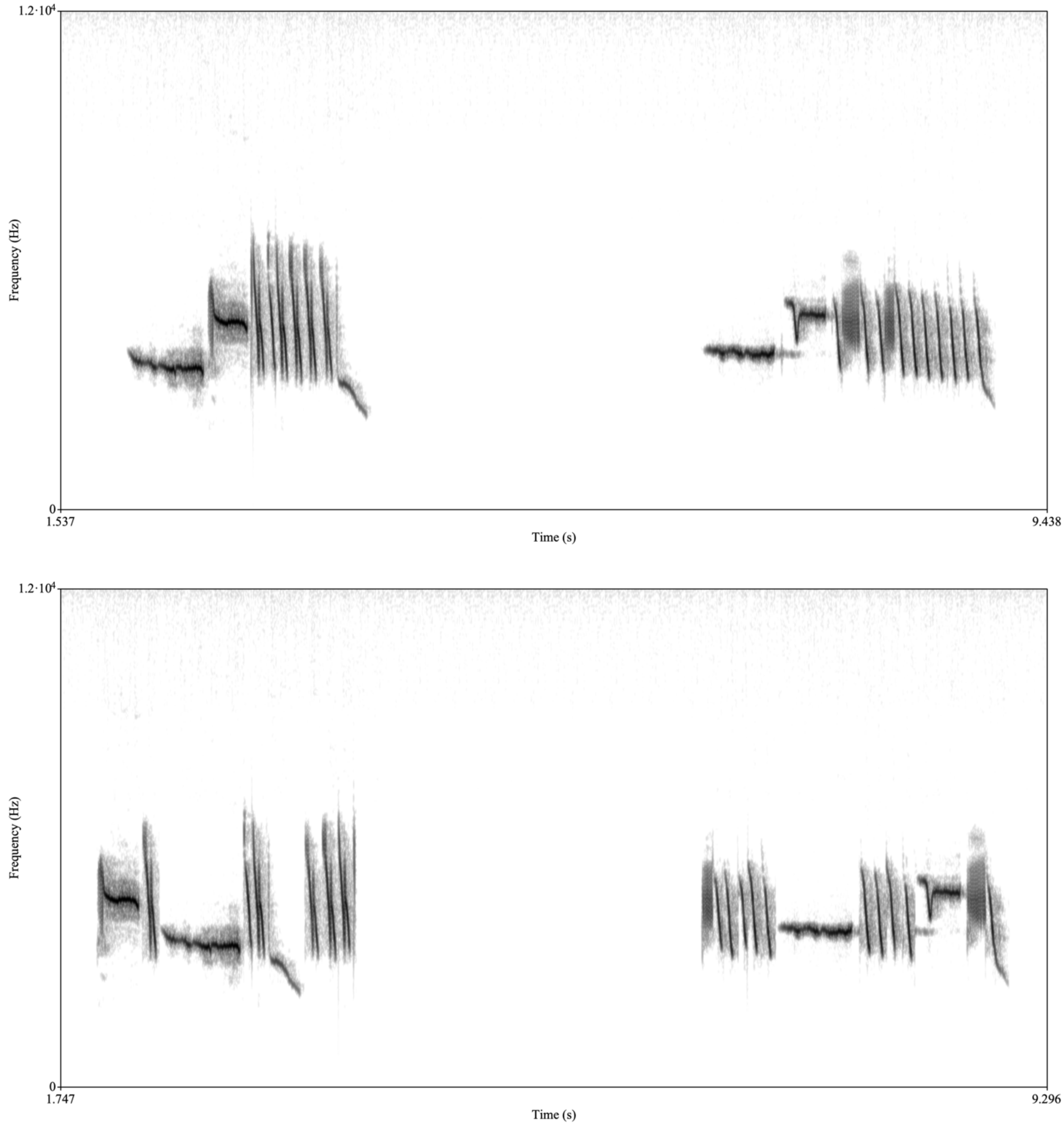

Supplementary Figure 4. Spectrogram representation of White-crowned Sparrow (*Zonotrichia leucophrys*) stimuli. Top, original element order; bottom, randomized element order.

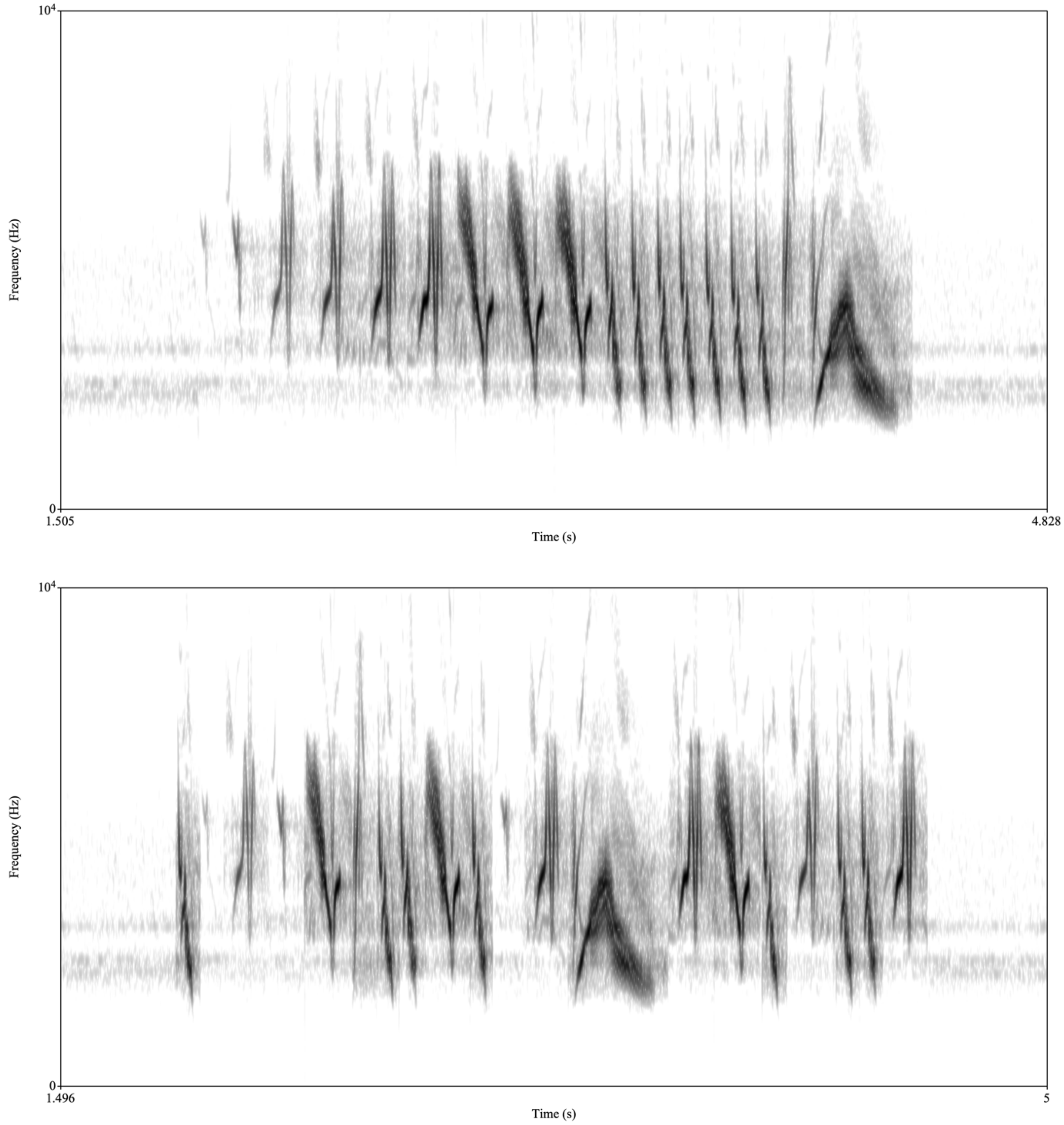

Supplementary Figure 5. Spectrogram representation of Common Chaffinch (*Fringilla coelebs*) stimuli. Top, original element order; bottom, randomized element order.

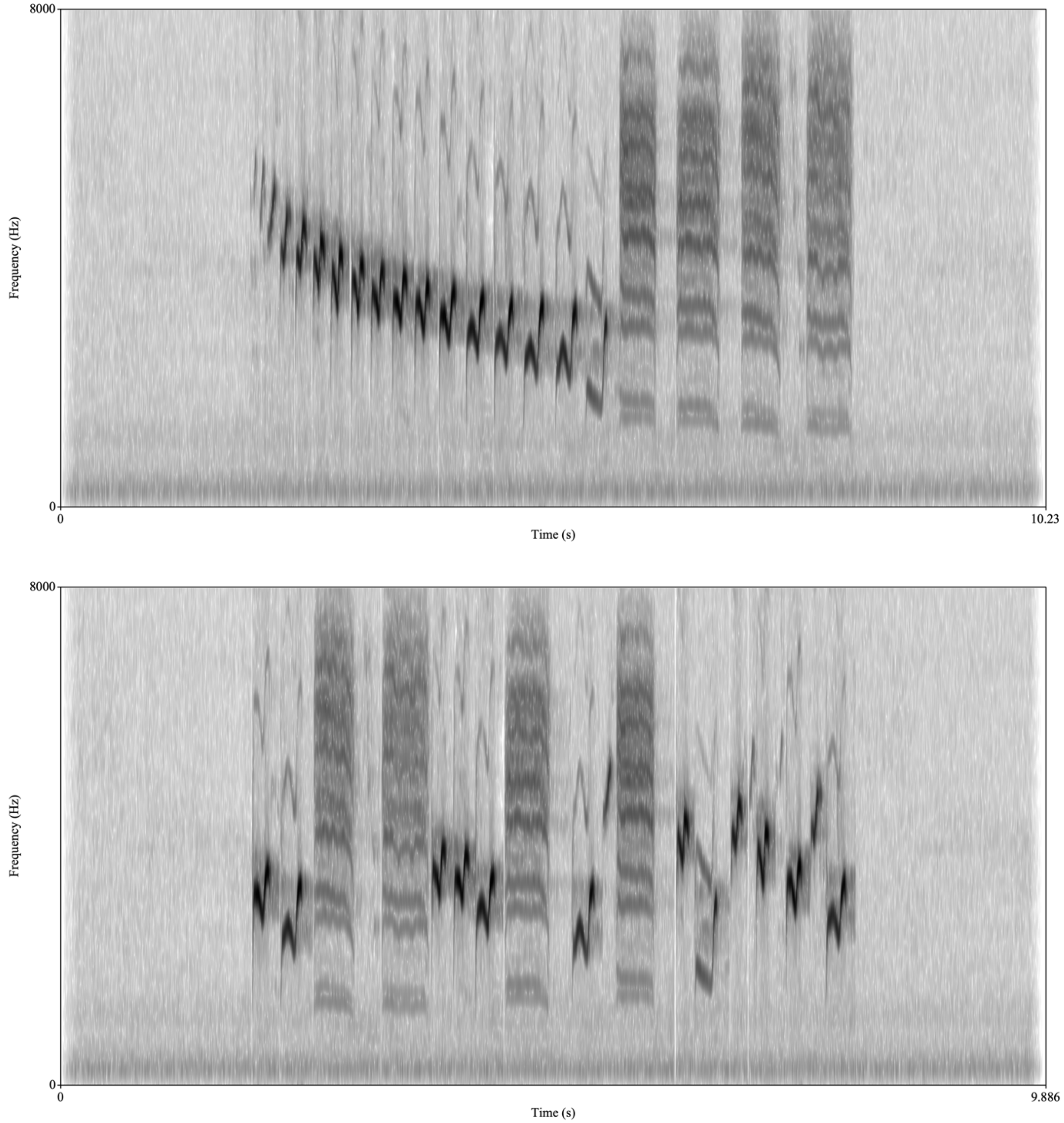

Supplementary Figure 6. Spectrogram representation of Canyon Wren (*Catherpes mexicanus*) stimuli. Top, original element order; bottom, randomized element order.

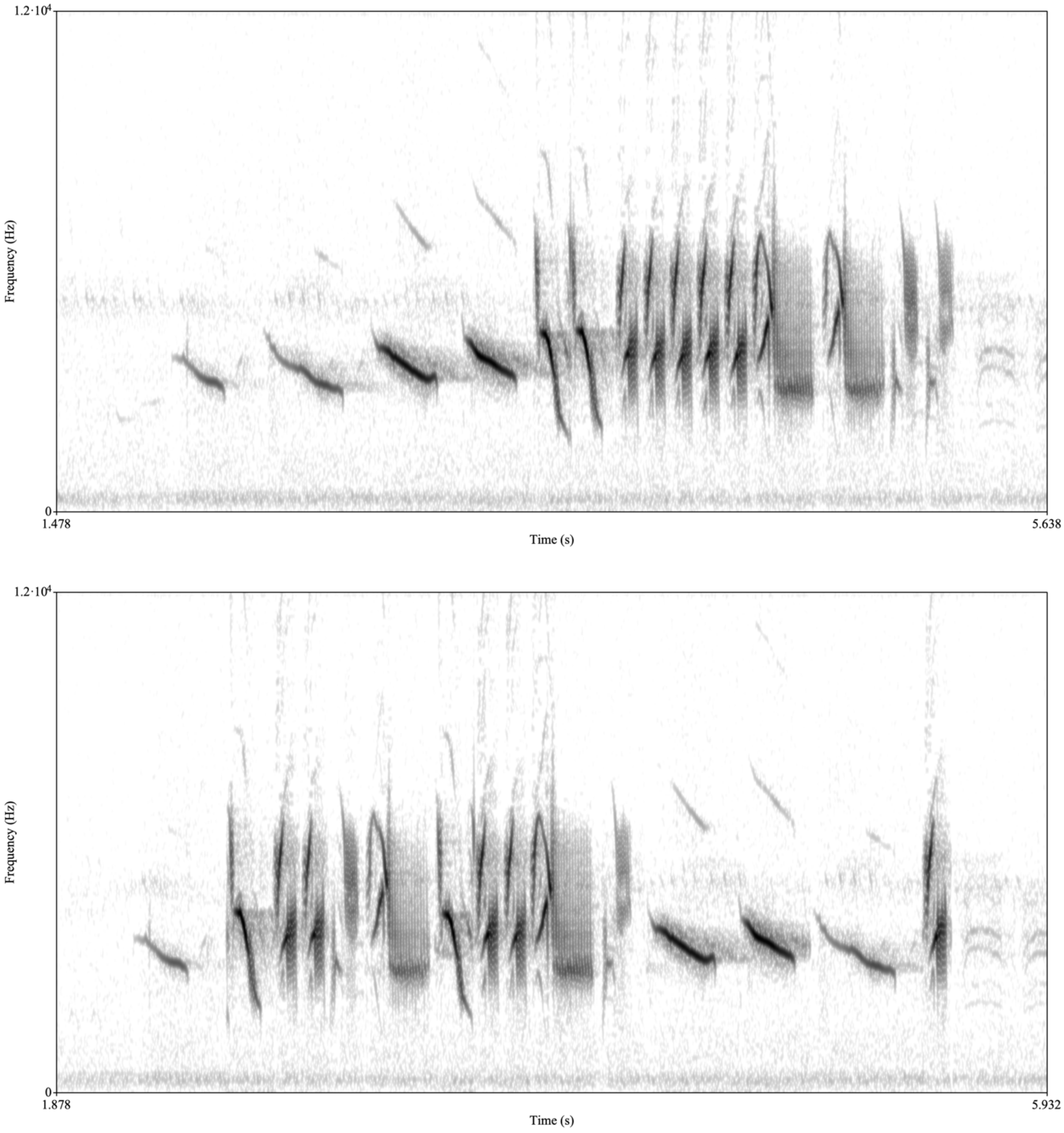

Supplementary Figure 7. Spectrogram representation of Vesper Sparrow (*Pooecetes gramineus*) stimuli. This was the first of three songs used in the study stimulus. Top, original element order; bottom, randomized element order.

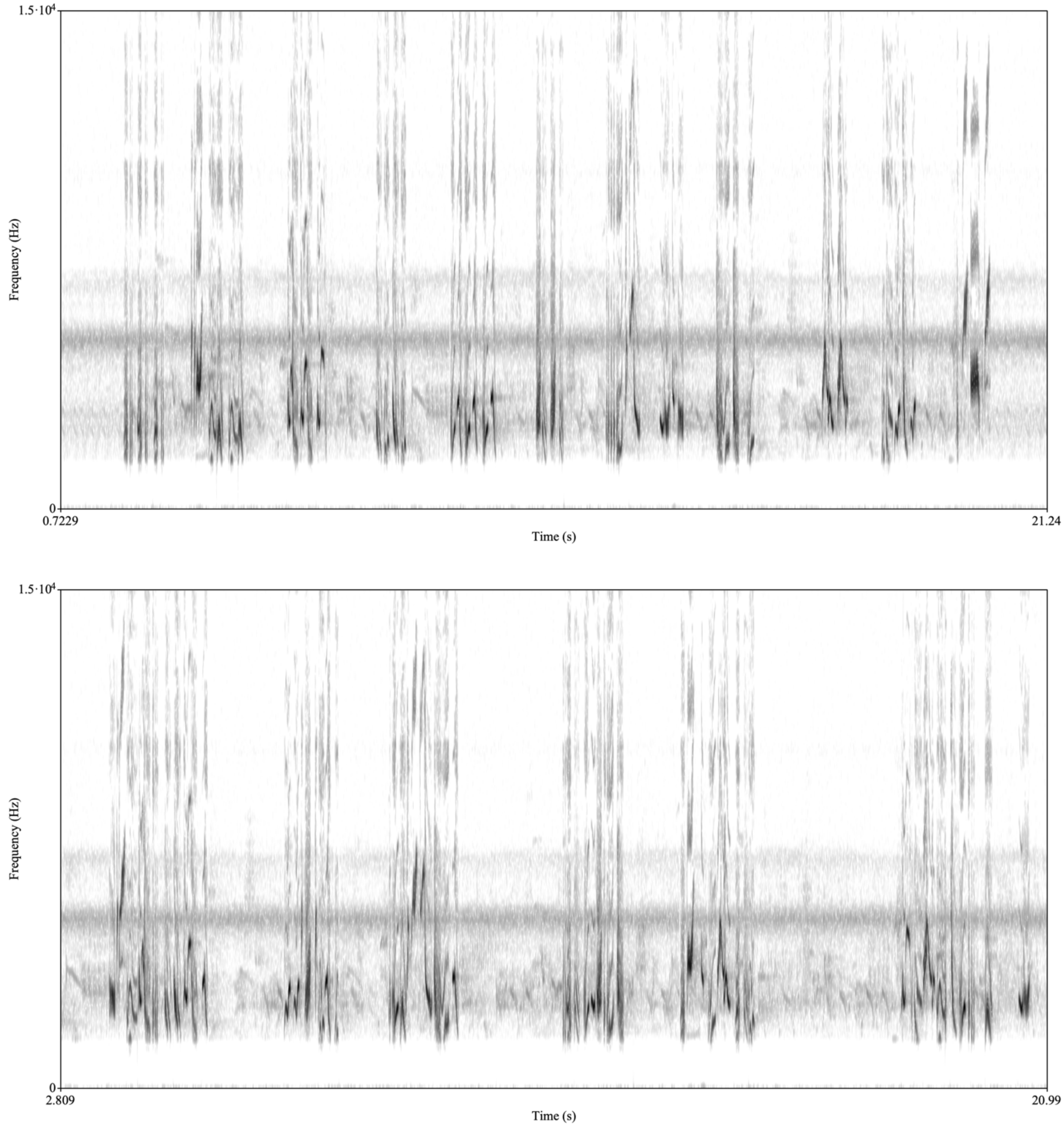

Supplementary Figure 8. Spectrogram representation of Brown Thrasher (*Toxostoma rufum*) stimuli. Top, original element order; bottom, randomized element order.

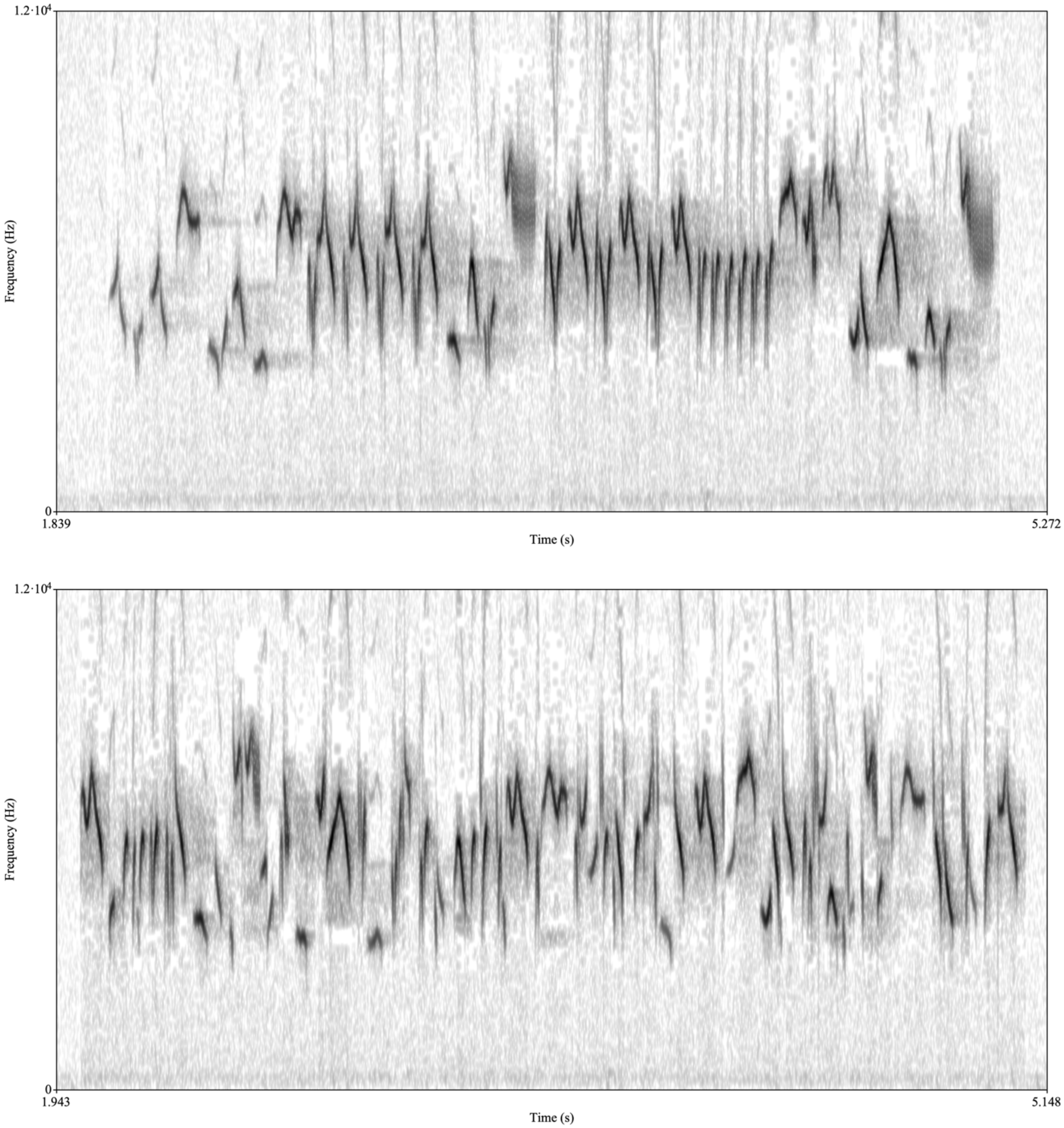

Supplementary Figure 9. Spectrogram representation of Winter Wren (*Troglodytes hiemalis*) stimuli. Top, original element order; bottom, randomized element order.

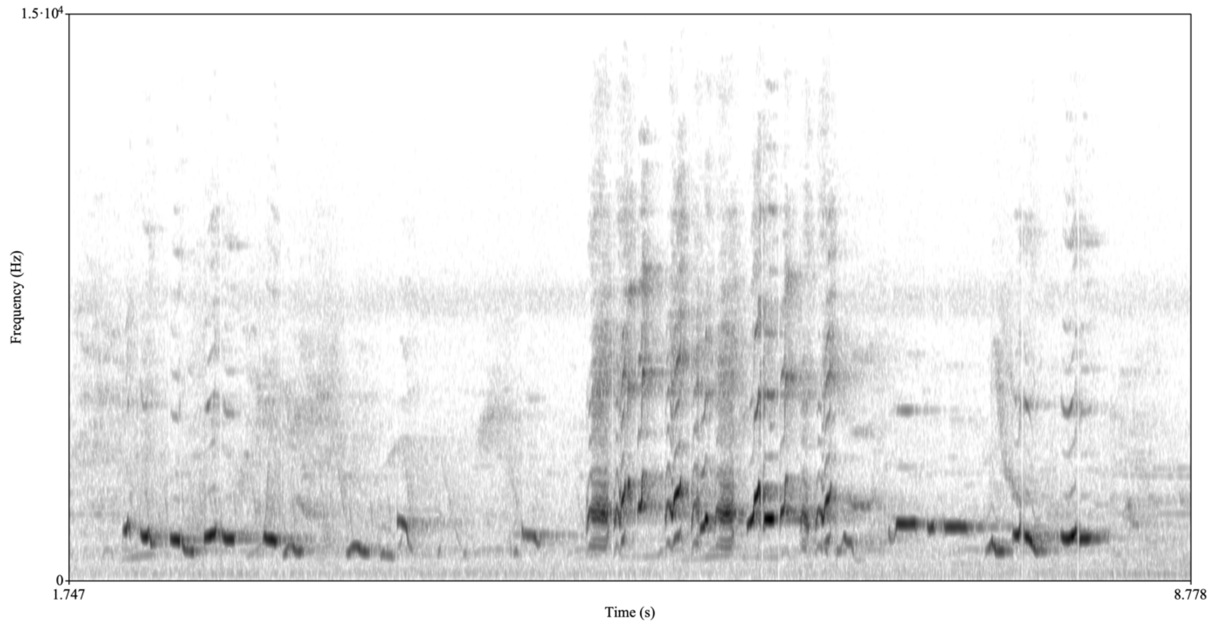

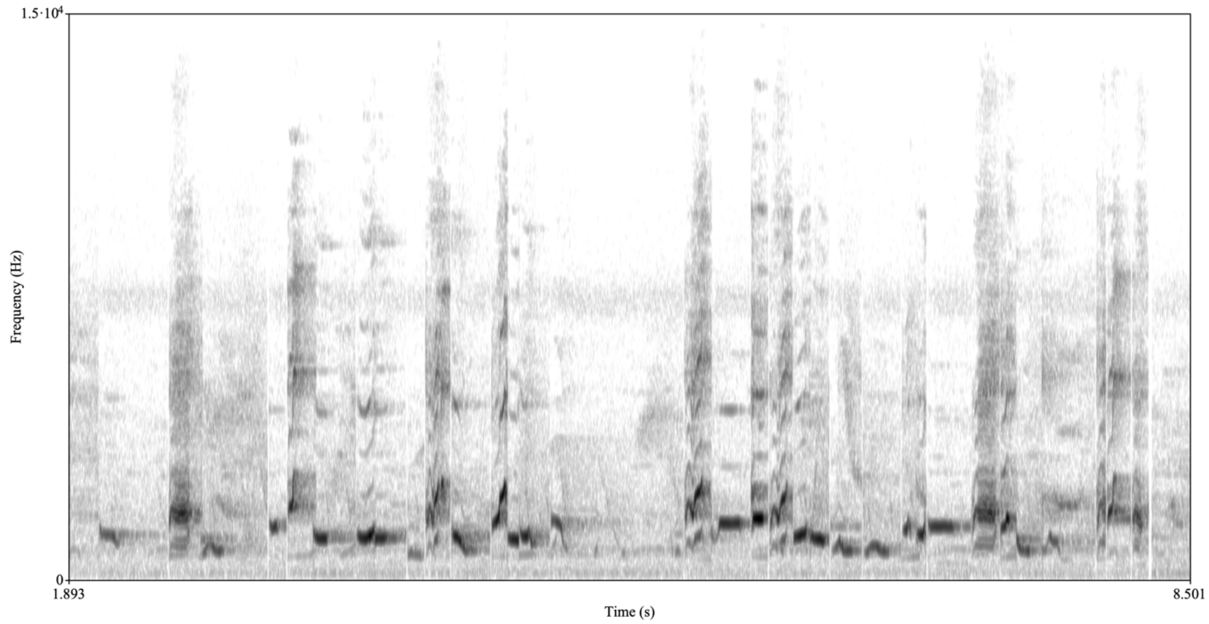

Supplementary Figure 10. Spectrogram representation of Grey Butcherbird (*Cracticus torquatus*) stimuli. This was the first of two songs used in the study stimulus. Top, original element order; bottom, randomized element order.

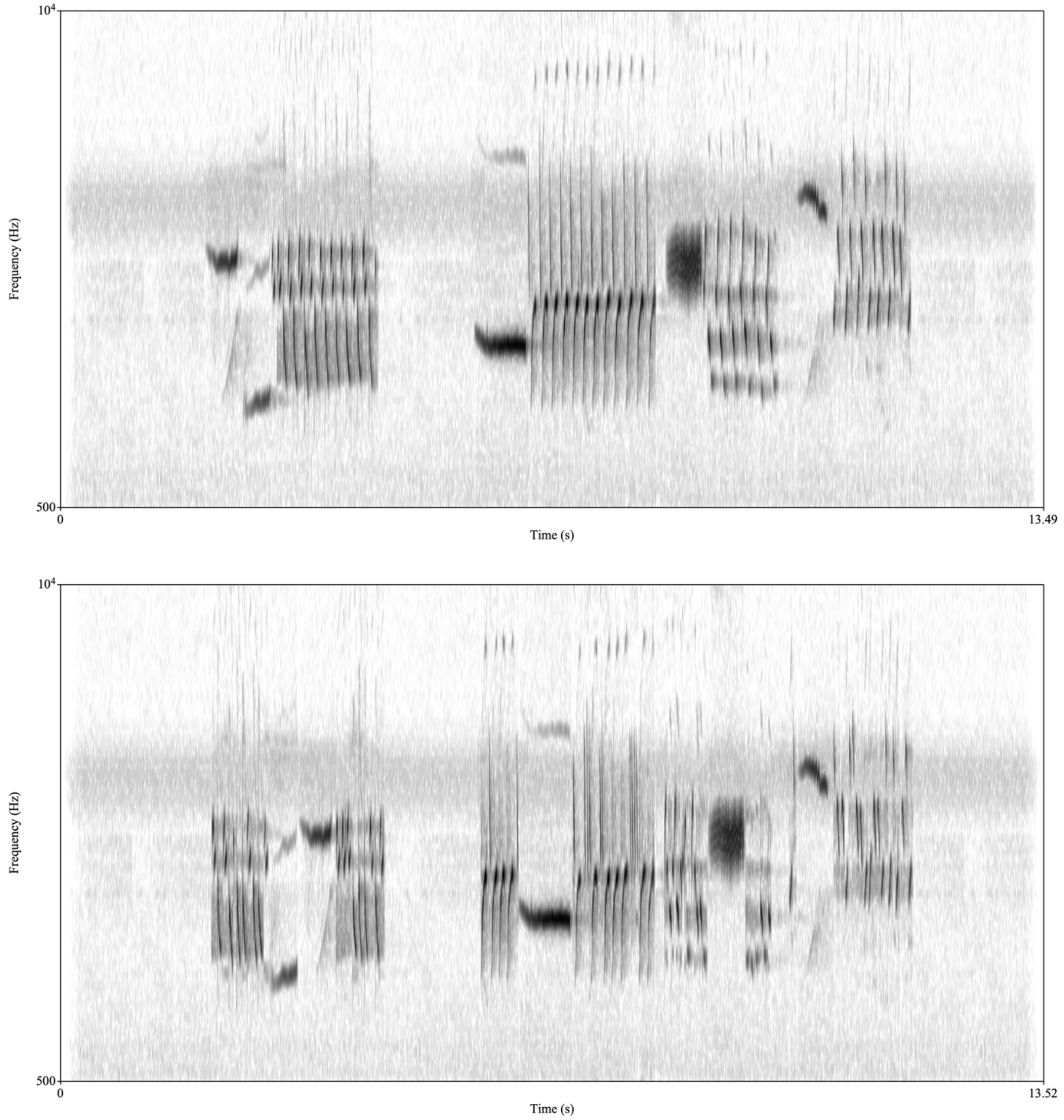

Supplementary Figure 11. Spectrogram representation of Bachman's Sparrow (*Peucaea aestivalis*) stimuli. Top, original element order; bottom, randomized element order.

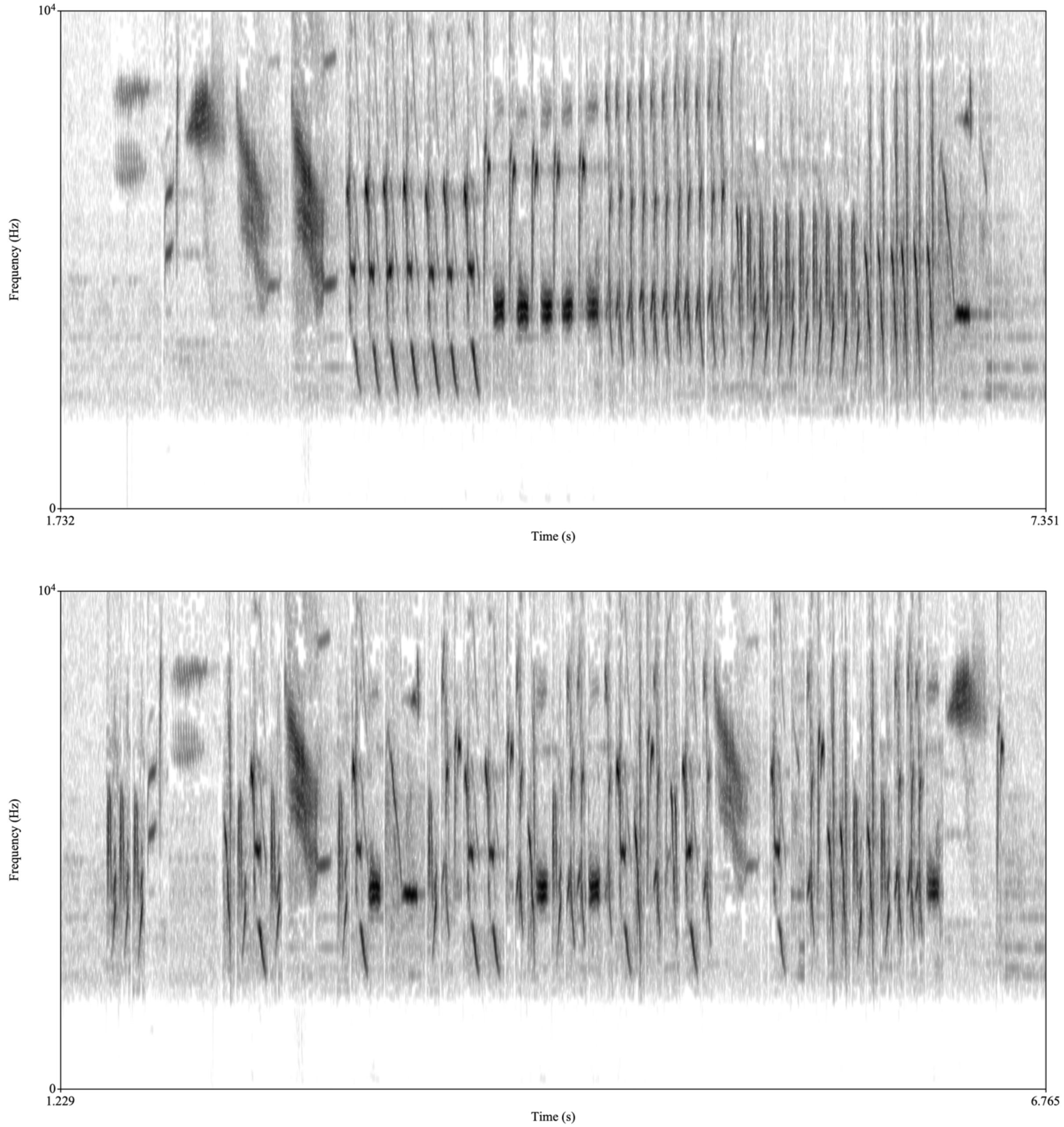

Supplementary Figure 12. Spectrogram representation of Citril Finch (*Carduelis citrinella*) stimuli. This was the first of three songs used in the study stimulus. Top, original element order. Bottom, randomized element order.

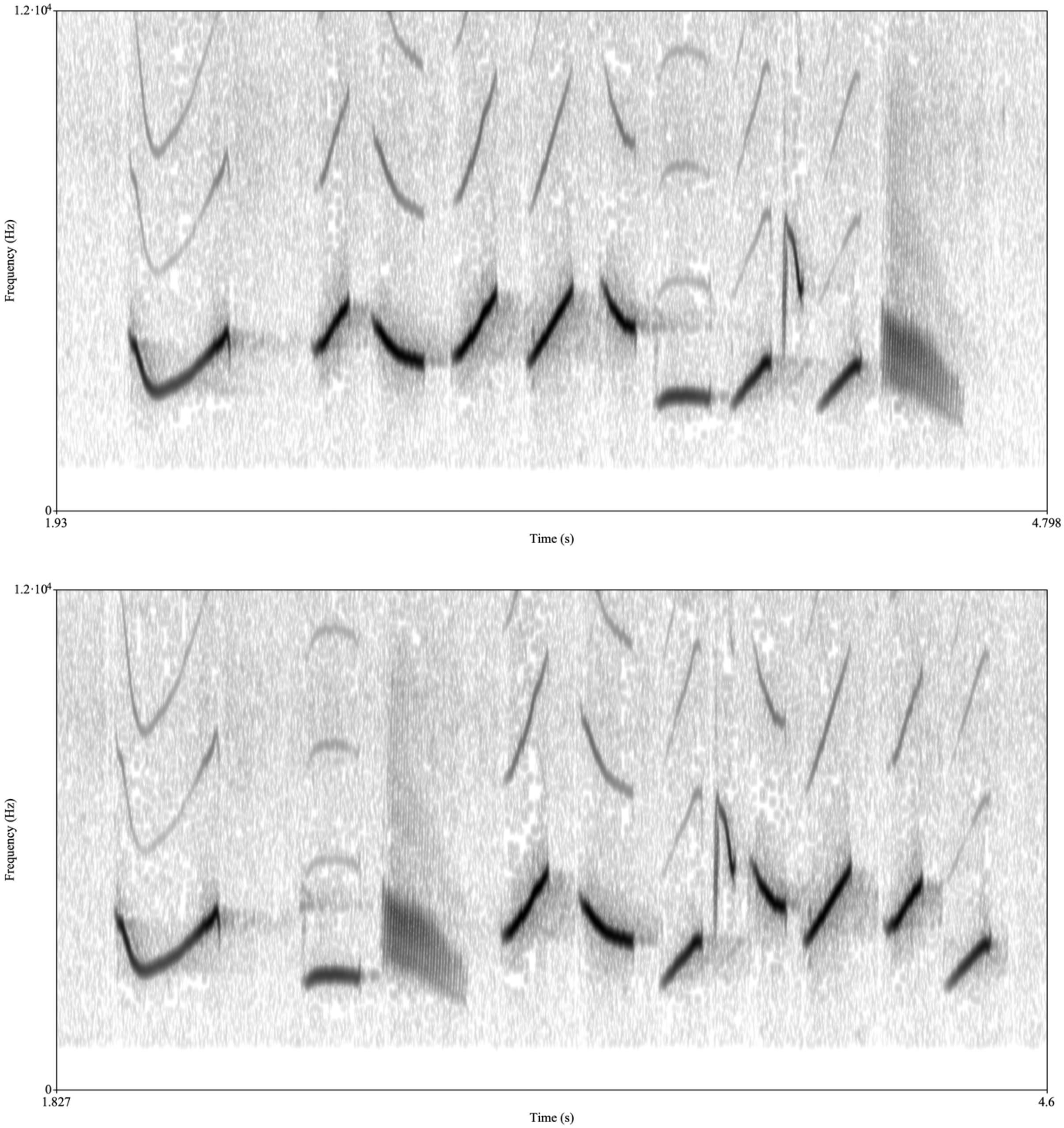

Supplementary Figure 13. Spectrogram representation of Red Fox-sparrow (*Passerella iliaca*) stimuli. Top, original element order; bottom, randomized element order.

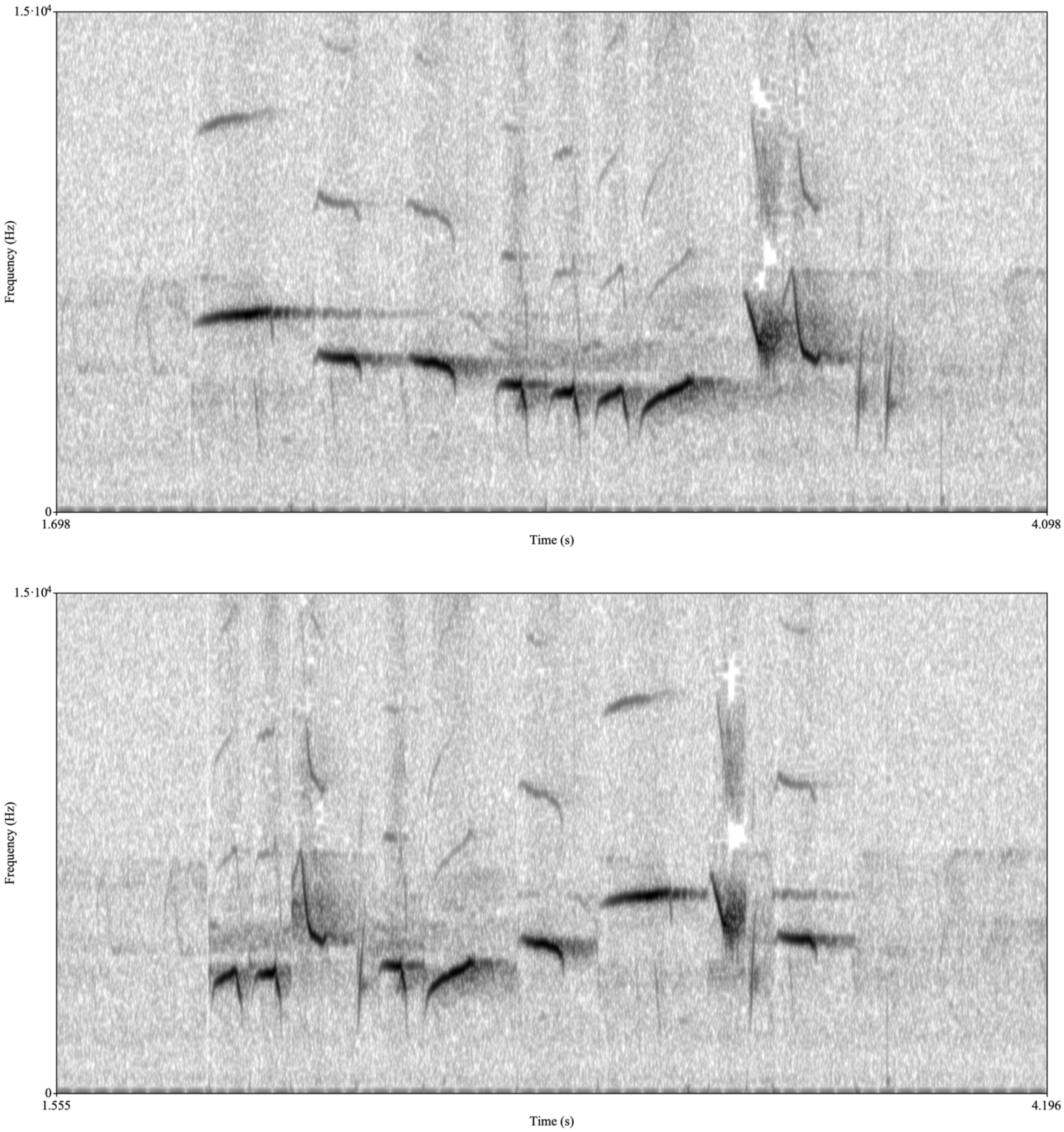

Supplementary Figure 14. Spectrogram representation of Madagascar Magpie-robin (*Copsychus albospecularis*) stimuli. This was the first of four songs used in the study stimulus. Top, original element order; bottom, randomized element order.

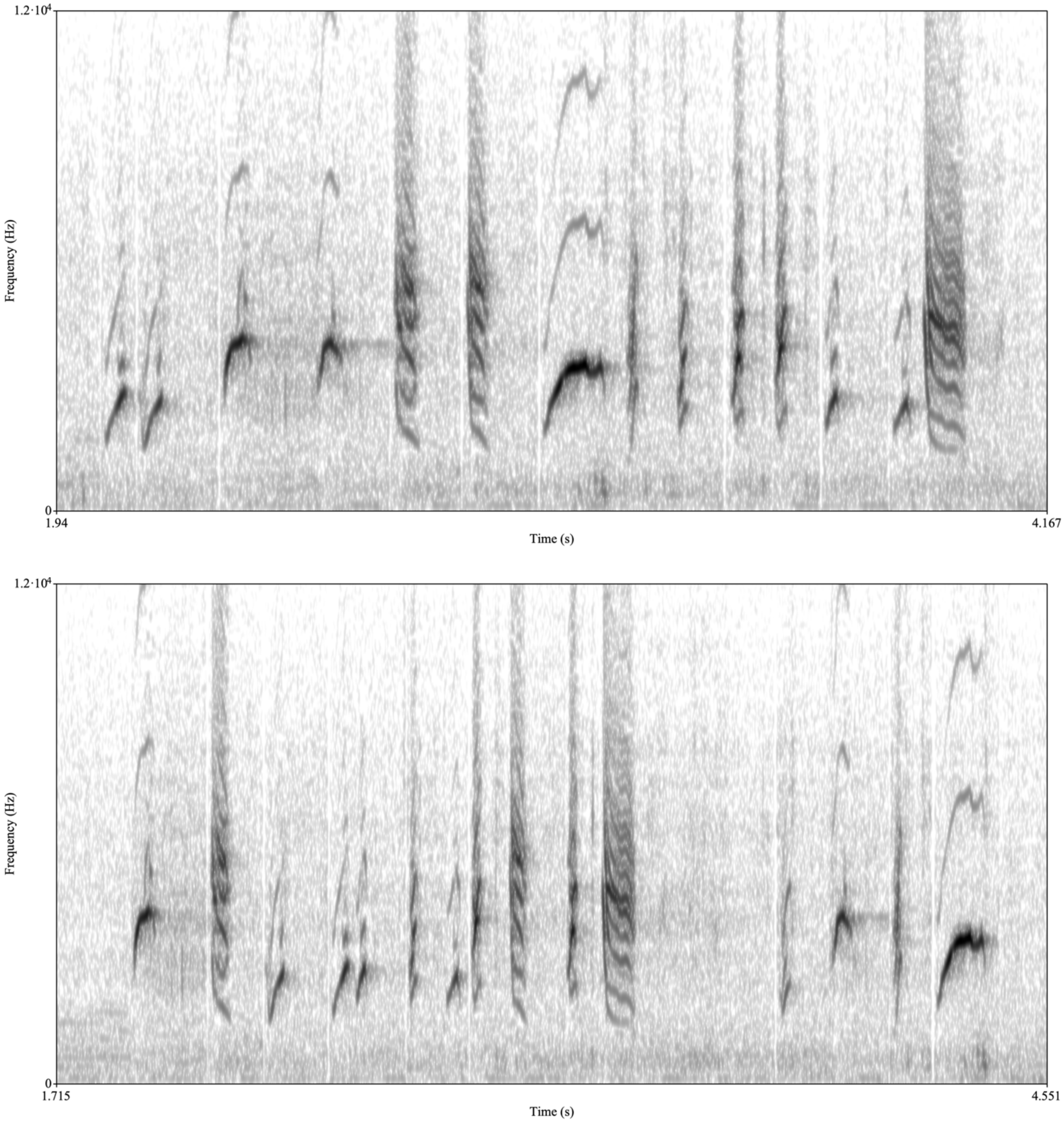

Supplementary Figure 15. Spectrogram representation of Striated Fieldwren (*Calamanthus fuliginosis*) stimuli. Top, original element order; bottom, randomized element order.

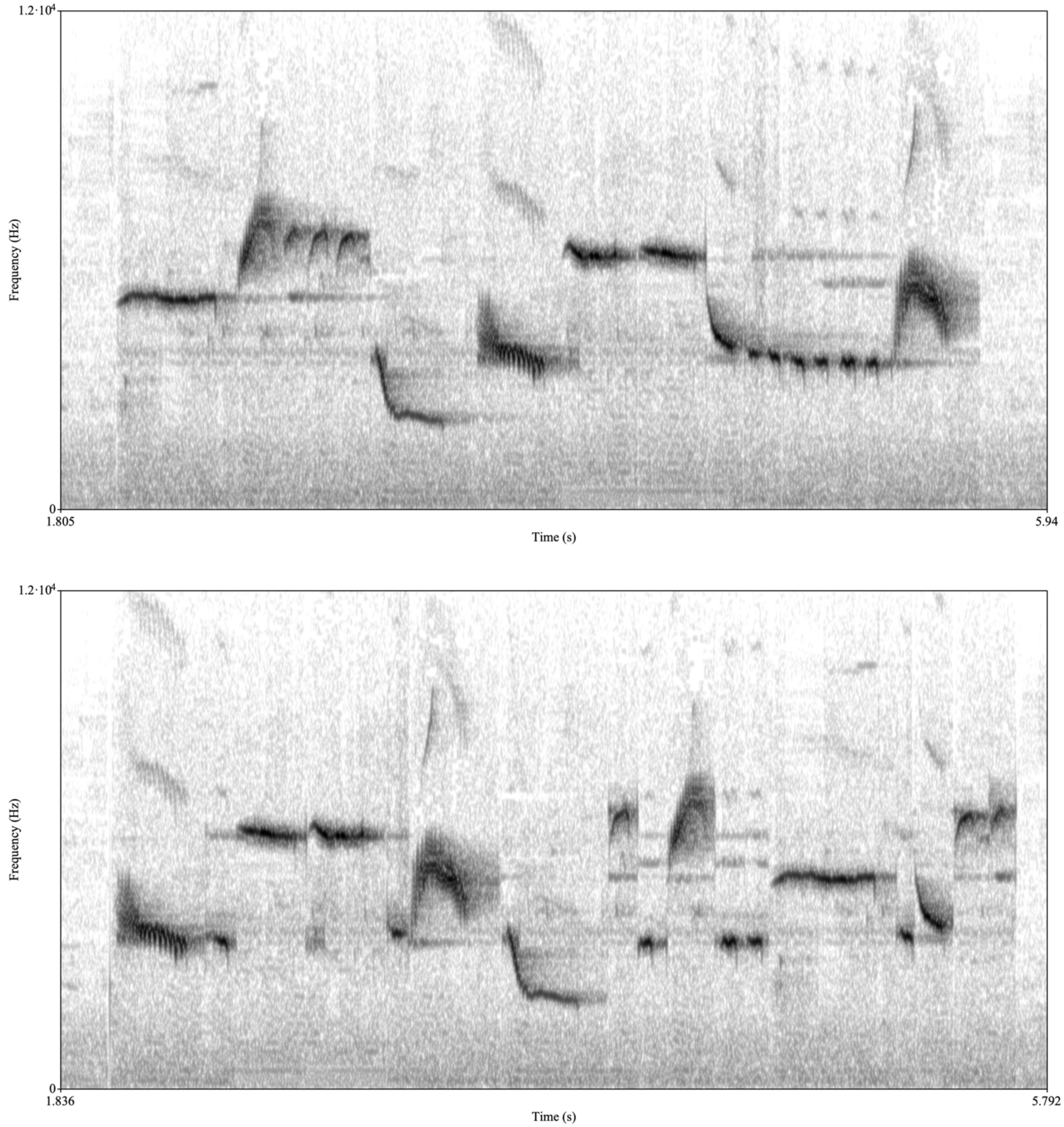

Supplementary Figure 16. Spectrogram representation of Scrubtit (*Acanthornis magna*) stimuli. Top, original element order; bottom, randomized element order.

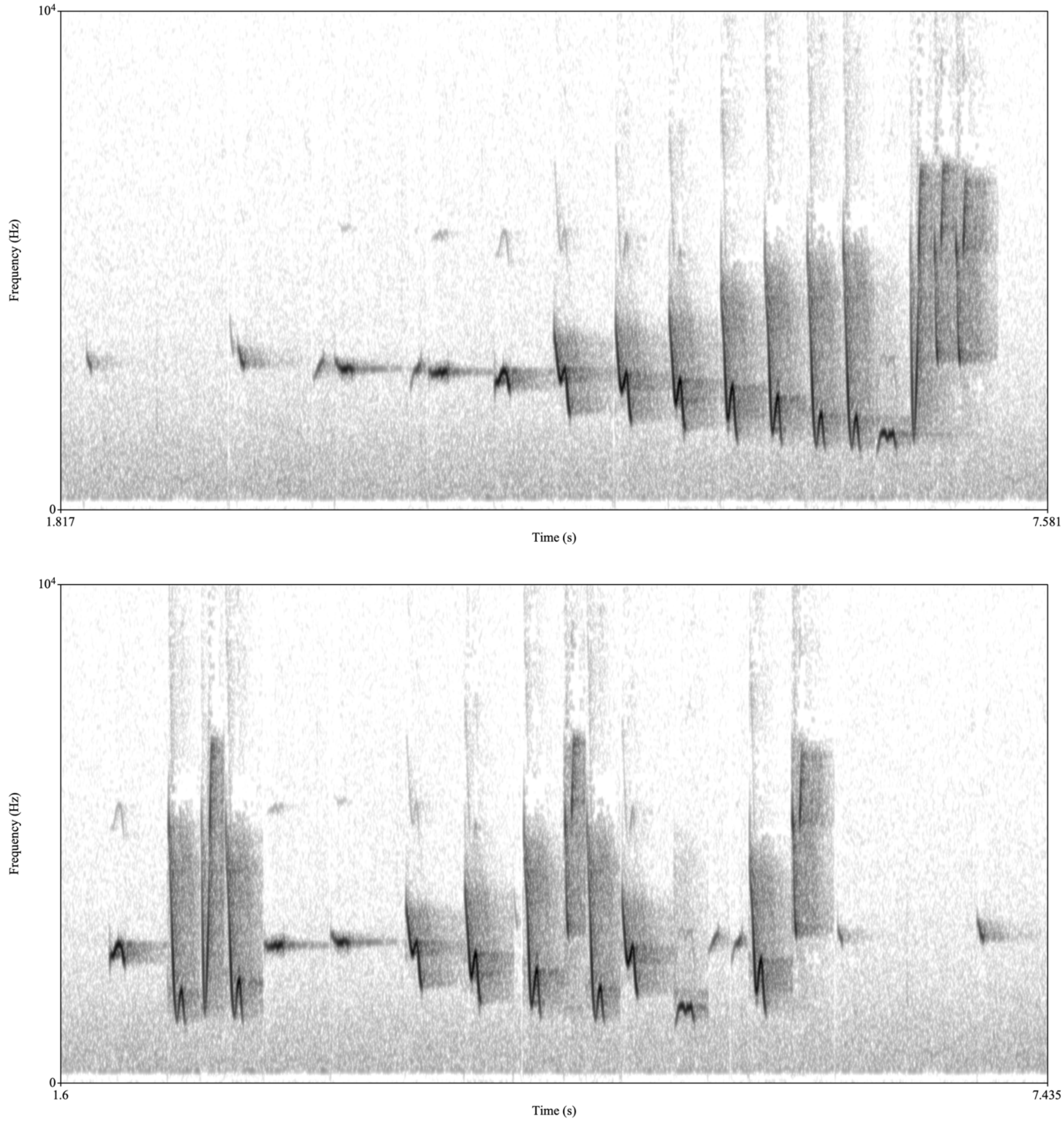

Supplementary Figure 17. Spectrogram representation of Noisy Scrub-bird (*Atrichornis clamosus*) stimuli. Top, original element order; bottom, randomized element order.

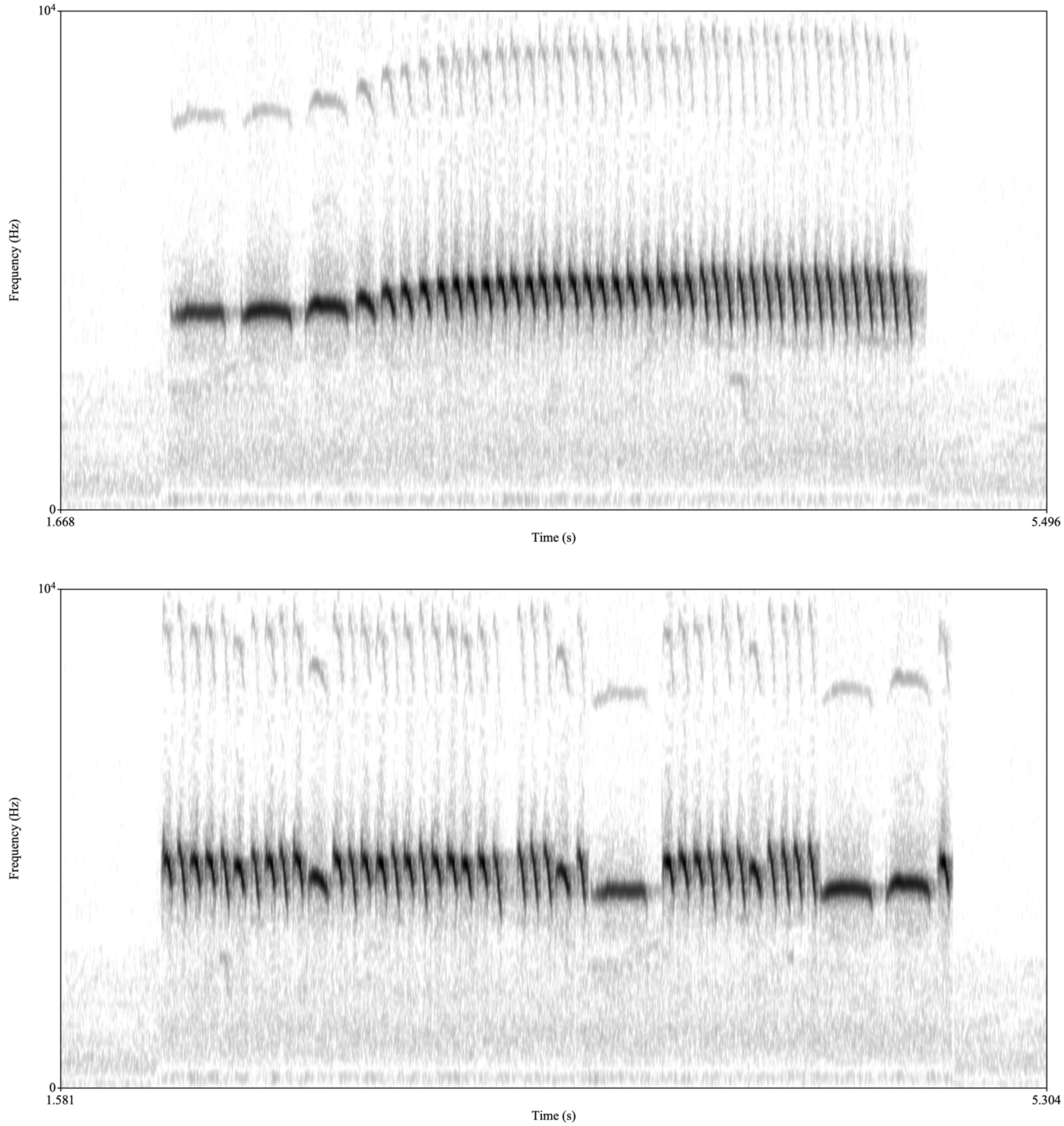

Supplementary Figure 18. Spectrogram representation of Field Sparrow (*Spizella pusilla*) stimuli. Top, original element order; bottom, randomized element order.

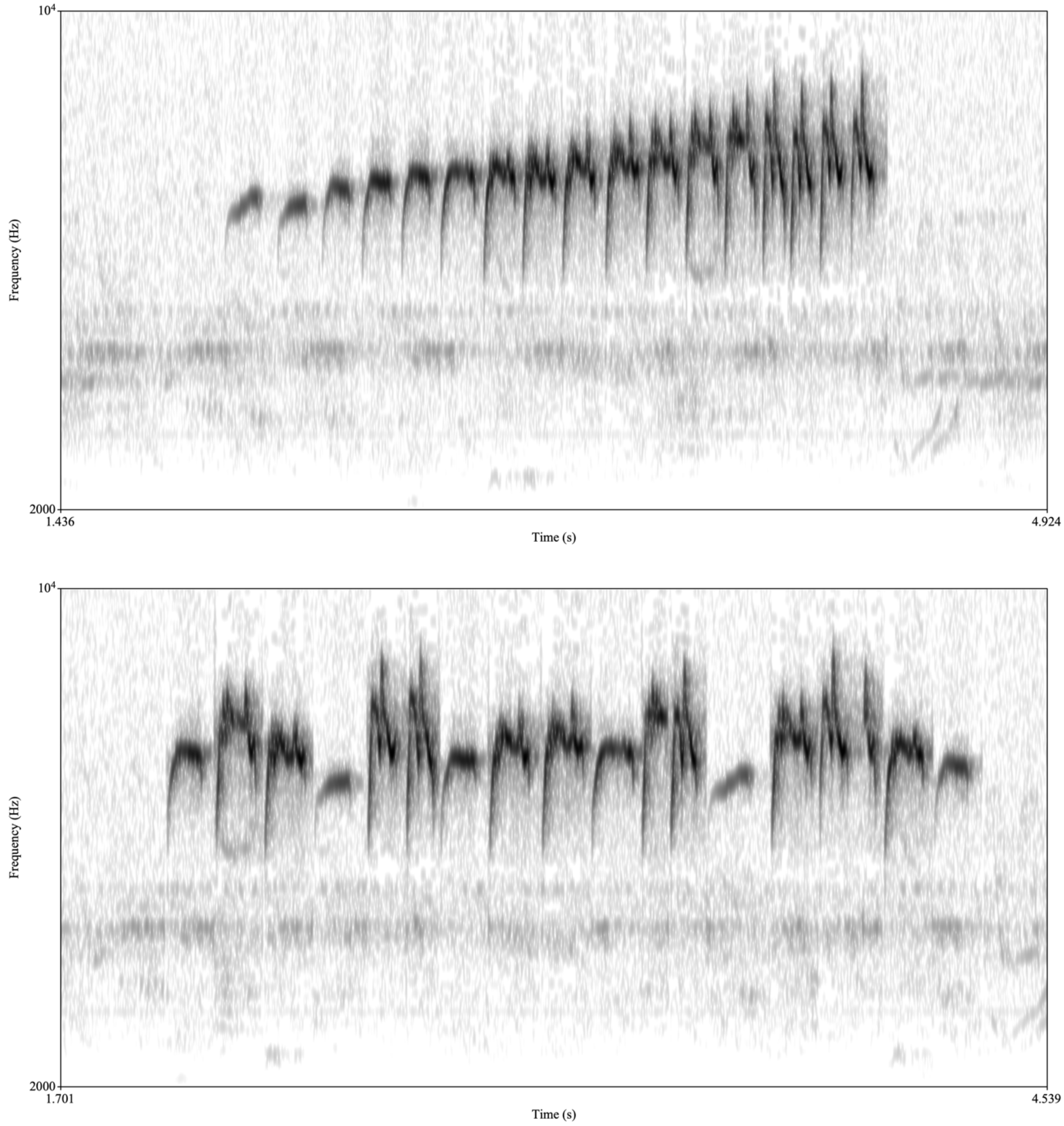

Supplementary Figure 19. Spectrogram representation of Common Firecrest (*Regulus ignicapilla*) stimuli. Top, original element order; bottom, randomized element order.

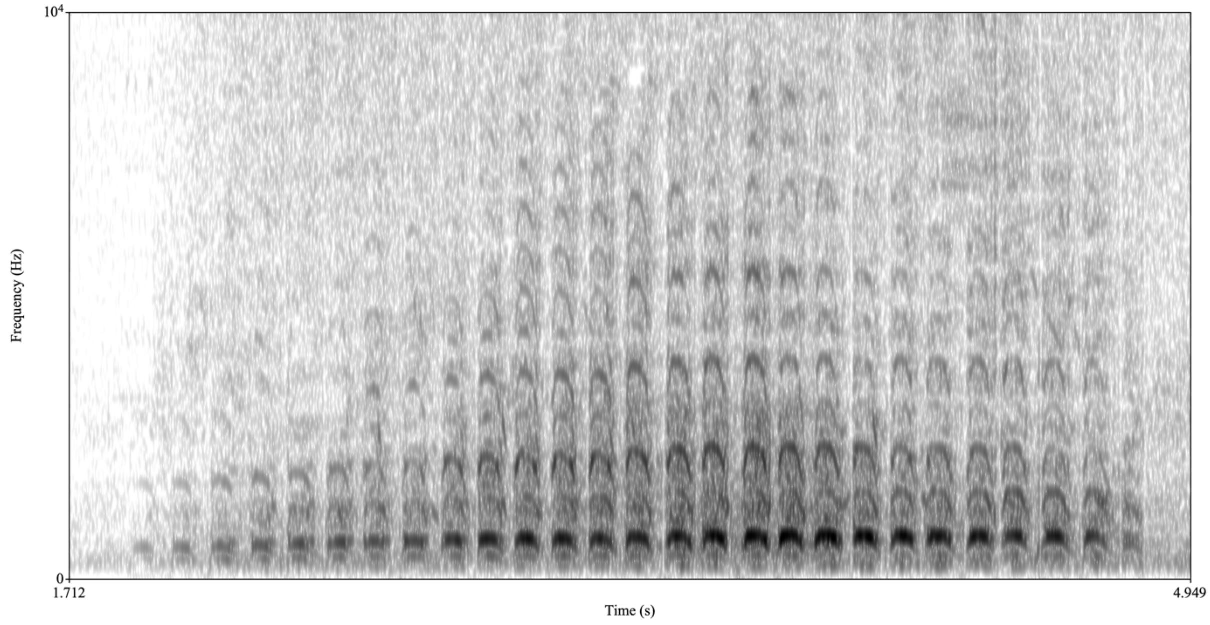

Supplementary Figure 20. Spectrogram representation of Wilson's Snipe (*Gallinago delicata*) stimuli. Top, original element order; bottom, randomized element order.

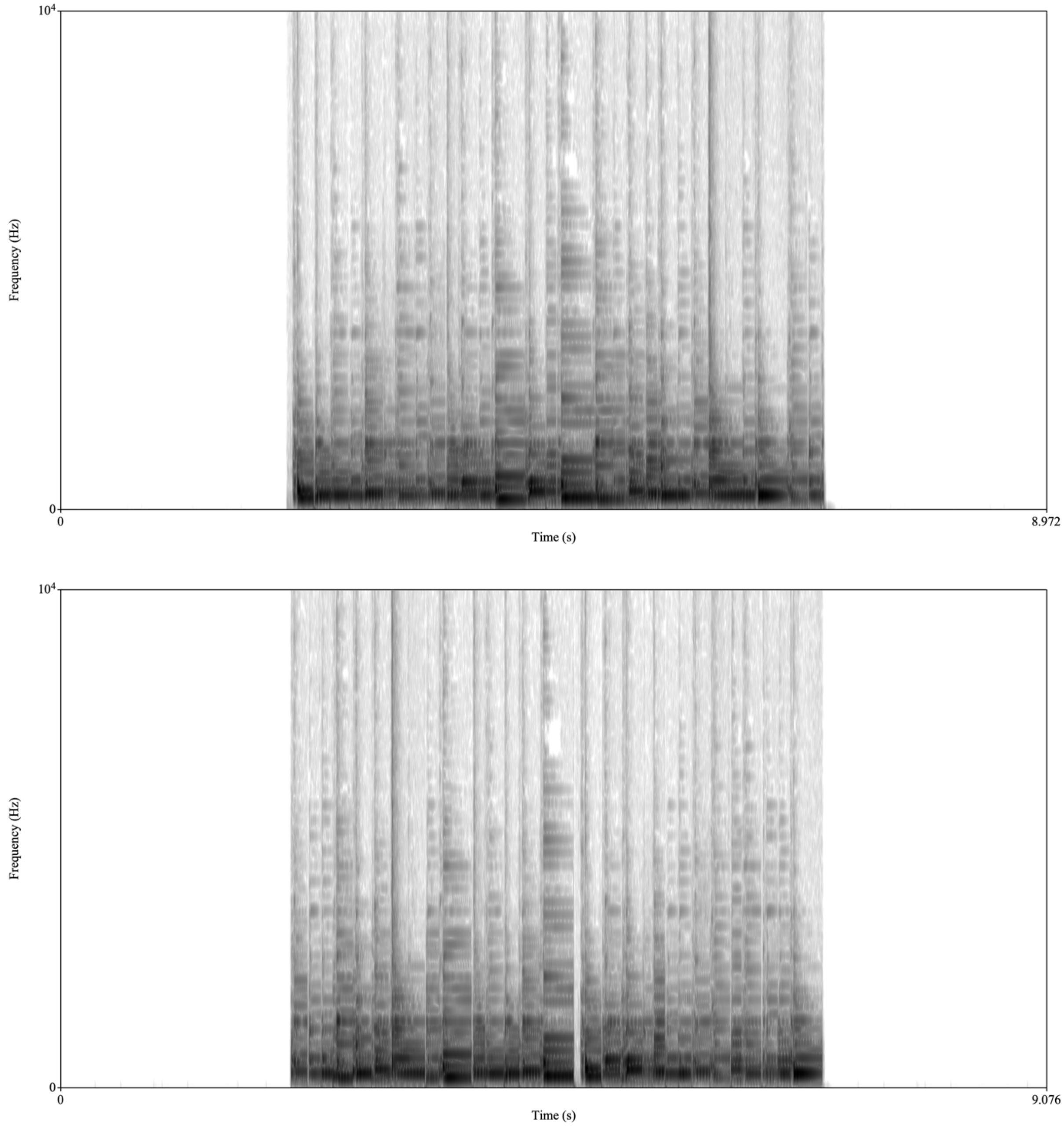

Supplementary Figure 21. Spectrogram representation of Human banjo music stimuli (one of the experiment's two musical controls). Top, original element order; bottom, randomized element order.

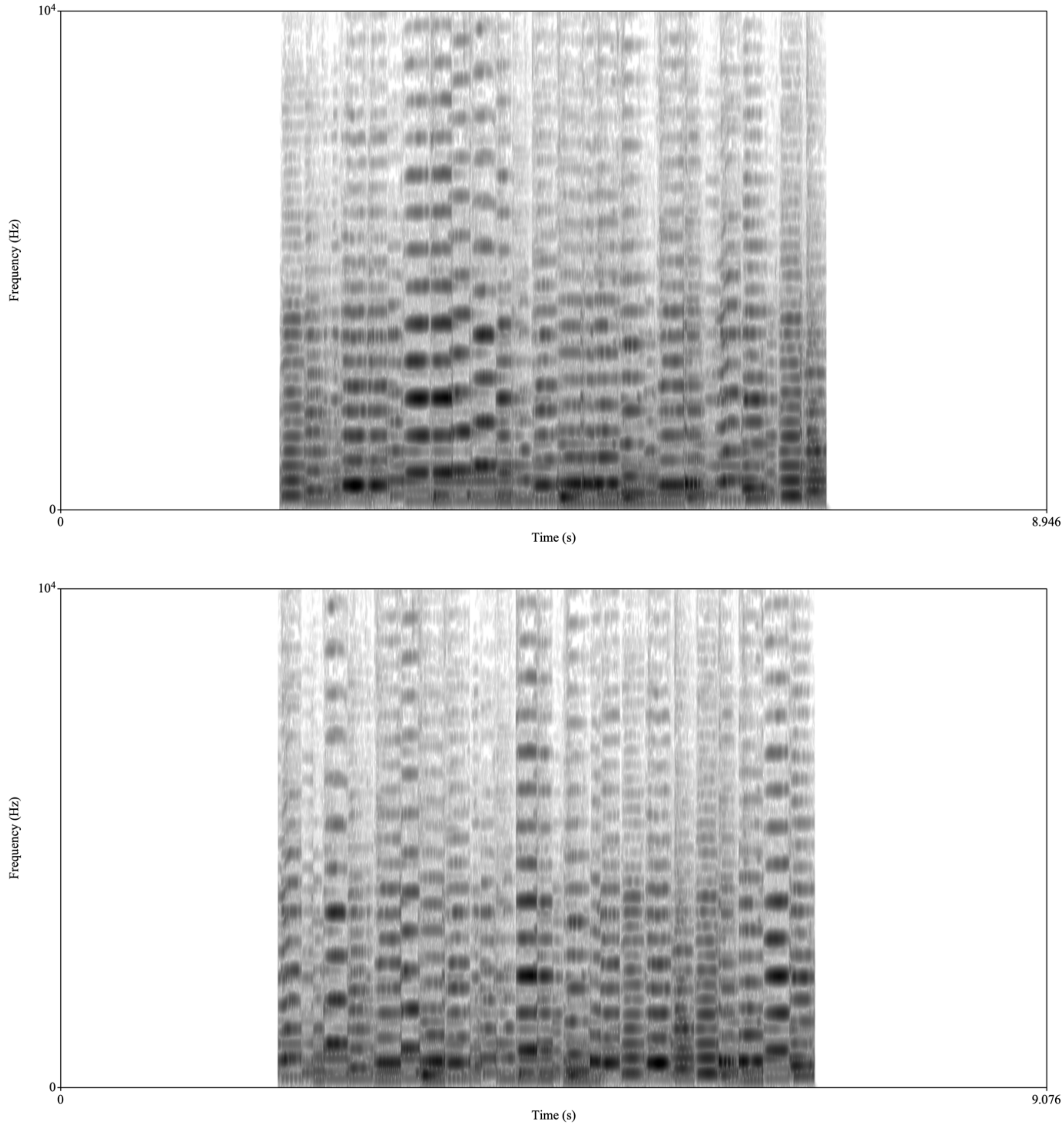

Supplementary Figure 22. Spectrogram representation of Human fiddle music stimuli (one of the experiment's two musical controls). Top, original element order; bottom, randomized element order.
